# Joint associations of leisure-time physical activity and daily sitting time with mortality among US adults classified as CKM stages 0–3: a prospective cohort study of NHANES 2007–2018

**DOI:** 10.1265/ehpm.26-00088

**Published:** 2026-10-20

**Authors:** Sufang Xu, Jiaming Huang, Lingrong Hu, Xiaoming Li, Shumin Zhao, Kaihong Chen, Liling Chen

**Affiliations:** Department of Cardiology, Longyan First Affiliated Hospital of Fujian Medical University, Longyan, 364000, China

**Keywords:** Cardiovascular-kidney-metabolic health, Daily sitting time, Leisure-time physical activity, Mortality, NHANES

## Abstract

**Background:**

Prior studies have linked physical activity to cardiovascular-kidney-metabolic (CKM) prevalence or mortality and sedentary behavior to CKM severity, and both behaviors are established mortality risk factors in the general population; however, prospective evidence on their joint associations with cause-specific mortality across CKM stages 0–3 remains limited.

**Methods:**

We analyzed 8,556 NHANES 2007–2018 participants aged ≥20 years with CKM stages 0–3, complete prespecified covariates, and a positive fasting-subsample weight. Leisure-time physical activity (LTPA) was categorized as inactive (0 min/week), insufficient (>0 to <150 min/week), or sufficient (≥150 min/week); daily sitting time was categorized as <6, 6 to <8, or ≥8 h/day. The primary outcome was all-cause mortality; cardiovascular disease (CVD) and non-CVD mortality were secondary outcomes. Survey-design-based Cox models incorporated the 12-year fasting weight, strata, and primary sampling units. Multiplicative and additive interactions were assessed.

**Results:**

During a median follow-up of 6.75 years, 670 deaths occurred, including 175 CVD and 495 non-CVD deaths. In fully adjusted models, sufficient versus no LTPA was associated with lower all-cause (hazard ratio [HR], 0.630; 95% confidence interval [CI], 0.483–0.822), CVD (HR, 0.536; 95% CI, 0.297–0.969), and non-CVD mortality (HR, 0.662; 95% CI, 0.478–0.916). Sitting ≥8 versus <6 h/day was associated with higher all-cause (HR, 1.506; 95% CI, 1.253–1.810), CVD (HR, 1.845; 95% CI, 1.246–2.732), and non-CVD mortality (HR, 1.406; 95% CI, 1.109–1.782). Compared with sufficient LTPA and sitting <6 h/day, <150 min/week of LTPA combined with sitting ≥8 h/day was associated with all-cause (HR, 1.972; 95% CI, 1.429–2.721), CVD (HR, 2.493; 95% CI, 1.232–5.045), and non-CVD mortality (HR, 1.806; 95% CI, 1.202–2.712). Multiplicative interaction was not detected for all-cause (P = 0.951) or non-CVD mortality (P = 0.274); the CVD interaction signal (P = 0.016) was exploratory because it depended on a joint category with few events.

**Conclusion:**

Higher LTPA and shorter sitting time were independently associated with lower mortality among US adults with CKM stages 0–3. Joint categories identified a higher-risk behavioral pattern, but did not establish that LTPA offsets the risk of prolonged sitting.

**Supplementary information:**

The online version contains supplementary material available at https://doi.org/10.1265/ehpm.26-00088.

## Introduction

Cardiovascular-kidney-metabolic (CKM) health integrates metabolic risk, kidney dysfunction, and cardiovascular disease along a staged continuum [1–3]. Briefly, stage 0 denotes optimal cardiometabolic health with no CKM risk factors; stage 1 reflects excess or dysfunctional adiposity (overweight, obesity, or abdominal obesity) or prediabetes; stage 2 comprises established metabolic risk factors (hypertriglyceridemia, hypertension, diabetes, or metabolic syndrome) or moderate-to-high-risk chronic kidney disease; and stage 3 indicates very-high-risk chronic kidney disease or a high predicted 10-year cardiovascular disease risk, corresponding to subclinical cardiovascular disease. Stage 0 therefore denotes optimal cardiometabolic health rather than disease, and stages 1–3 represent progressively greater metabolic, kidney, or subclinical cardiovascular risk. Accordingly, this study refers to adults classified as CKM stages 0–3 rather than “CKM syndrome patients.” CKM risk factors and chronic kidney disease are common and are associated with substantial cardiovascular and mortality burdens [4, 5].

Sedentary behavior—defined as activities with energy expenditure less than or equal to 1.5 metabolic equivalents while in a sitting or reclining posture—has emerged as a significant public health concern [6, 7]. The pervasiveness of sedentary lifestyles, particularly in industrialized societies, has evolved in parallel with technological advancements that reduce physical demands in daily activities. Growing evidence suggests that prolonged sedentary time is independently associated with adverse health outcomes, demonstrating clear dose-response relationships with cardiovascular and all-cause mortality [8–10]. This association persists even after adjusting for moderate-to-vigorous physical activity, indicating that sedentary behavior represents a distinct health risk factor rather than merely an absence of exercise. A dose-response meta-analysis found that the association was nonlinear: overall mortality risk increased by approximately 2% per additional hour of sitting, while among adults sitting for >7 h/day, each additional hour was associated with an approximately 5% higher risk [11]. Given the ubiquity of sedentary behavior in contemporary society, this translates to substantial population-attributable risk. Likewise, in the prospective HUNT3 population cohort, higher sedentary time was associated with increased mortality from all causes and cardiometabolic diseases [12], and a meta-regression including more than one million participants identified a threshold of daily sedentary time beyond which all-cause mortality increased [13].

Previous evidence supports associations of physical activity and sedentary behavior with CKM-related outcomes, but it does not resolve the present question. In the general population, leisure-time physical activity accumulated across the adult life course has been associated with lower all-cause and cause-specific mortality [14]. Within the CKM literature, higher physical activity was associated with lower CKM prevalence among older Chinese adults; vigorous-volume physical activity was associated with lower all-cause mortality in a short follow-up study of Chinese adults with CKM; and an NHANES cross-sectional analysis related greater moderate-to-vigorous activity to less advanced CKM stage while finding weaker associations for sedentary behavior [15–17]. These studies evaluated CKM prevalence or stage, or physical activity alone, rather than the prospective joint associations of LTPA and sitting time with cause-specific mortality across CKM stages 0–3. Studies in general and selected clinical populations also indicate that sitting and physical activity may contribute information independently [10, 18–21]. Because CKM stages 0–3 encompass most of the adult population, these established associations are expected to apply to our cohort as well; what remains unclear is whether the two behaviors, examined jointly, identify distinct mortality-risk strata along the CKM continuum and whether absolute and cause-specific risks differ across stages.

We therefore evaluated the independent and joint associations of LTPA and daily sitting time with mortality among US adults classified as CKM stages 0–3. All-cause mortality was prespecified as the primary outcome, with CVD and non-CVD mortality as secondary outcomes. We further tested multiplicative and additive interaction, examined sitting-time associations within LTPA strata and LTPA associations within sitting-time strata, and assessed whether the findings were sensitive to CKM stage, PREVENT age restrictions, CKM-component missingness, and alternative covariate sets. We hypothesized that lower LTPA and longer sitting would each be associated with higher mortality; interaction analyses were treated as tests of effect modification rather than inferred from combined categories alone.

## Methods

### Study design and population

This prospective cohort analysis used six 2-year cycles of the National Health and Nutrition Examination Survey (NHANES), 2007–2018. NHANES uses a stratified, multistage probability sample of the US noninstitutionalized civilian population. Baseline interviews, examinations, and laboratory measurements were linked to public-use mortality follow-up through December 31, 2019 [22].

Participant selection is detailed in Fig. S1. Among 59,842 NHANES participants, 25,072 aged <20 years were excluded. Of 34,770 adults aged ≥20 years, 18,707 could not be classified because CKM information was insufficient and 1,920 with CKM stage 4 were excluded, leaving 14,143 classified as stages 0–3. We then excluded 9 participants with missing LTPA, 50 with missing sitting time, 36 without mortality follow-up, and 160 who were pregnant, yielding 13,888 exposure- and outcome-eligible participants. A further 4,510 with a missing value for at least one prespecified adjustment variable (age, sex, race/ethnicity, education, poverty-income ratio, alcohol use, or smoking) were excluded, leaving 9,378 complete cases. Finally, 822 participants with a zero fasting-subsample weight were excluded; the primary analytic cohort comprised 8,556 adults aged ≥20 years. Multiple imputation was not performed because the available updated file contained only complete cases; this complete-case restriction and the potential for selection bias were considered when interpreting generalizability.

### Definition of CKM syndrome stages 0–3

CKM stages were assigned hierarchically according to the 2023 American Heart Association advisory [3]. Stage 0 required normal BMI (<23 kg/m^2^ for Asian participants and <25 kg/m^2^ for all others), normal waist circumference, and no criterion for a higher stage. Stage 1 included excess adiposity (BMI ≥23 kg/m^2^ for Asian participants or ≥25 kg/m^2^ for all others), elevated waist circumference, or prediabetes. Stage 2 included metabolic risk factors—fasting triglycerides ≥135 mg/dL, hypertension, diabetes, or metabolic syndrome—or moderate/high-risk CKD according to estimated glomerular filtration rate (eGFR) and urinary albumin-to-creatinine ratio (UACR). Stage 3 included very-high-risk CKD or a PREVENT-predicted 10-year CVD risk ≥20%. Stage 4 (established CVD) was excluded. The corrected non-Asian BMI threshold of ≥25 kg/m^2^ removes the ambiguity at exactly 25 kg/m^2^. Full definitions are provided in Table S1.

Fasting glucose and triglycerides required the fasting subsample. Laboratory values were not imputed. Within the final cohort, missing values remained for BMI (n = 63), waist circumference (n = 182), glycated hemoglobin (n = 16), triglycerides (n = 77), HDL cholesterol (n = 75), eGFR (n = 105), and UACR (n = 58); fasting glucose was complete. The rule-based staging variable was retained when another observed criterion had assigned a stage, and a sensitivity analysis required all eight CKM components (n = 8,210). PREVENT (Predicting Risk of Cardiovascular Disease EVENTs) is the American Heart Association’s sex-specific risk-equation system that estimates the 10-year absolute risk of total cardiovascular disease—atherosclerotic cardiovascular disease and heart failure—in adults aged 30–79 years without known cardiovascular disease, using routinely measured clinical variables (age, total and HDL cholesterol, systolic blood pressure, blood-pressure-lowering and statin treatment, diabetes, smoking, and estimated glomerular filtration rate) [23]. PREVENT was calculated for ages 30–79 years; the 417 participants whose public-use age was top-coded at 80 were assigned age 79 for PREVENT only [23]. Stage 3 was attributable to PREVENT risk ≥20% without very-high-risk CKD in 612 participants; 116 had very-high-risk CKD, including 95 who also had PREVENT risk ≥20%. Values outside PREVENT-supported ranges were set to the nearest boundary: total cholesterol in 2,556 participants, HDL cholesterol in 117, systolic blood pressure in 72, and eGFR in 18; 2,722 participants had at least one truncated value. Cardiac biomarkers and cardiovascular imaging were unavailable. Sensitivity analyses excluded age-top-coded participants, restricted the cohort to CKM stages 0–2, and required complete CKM laboratory data.

### Assessment of leisure-time physical activity and daily sitting time

LTPA was assessed with the Global Physical Activity Questionnaire [24]. Weekly moderate-equivalent LTPA minutes were calculated as minutes of moderate-intensity recreational activity plus twice the minutes of vigorous-intensity recreational activity. Categories were inactive (0 min/week), insufficiently active (>0 to <150 min/week), and sufficiently active (≥150 min/week). The continuous estimate was scaled per additional 60 moderate-equivalent minutes per week (one moderate-equivalent hour/week).

Participants reported the total time they usually spent sitting or reclining on a typical day, excluding sleep. Reported minutes were converted to hours/day and categorized as <6, 6 to <8, or ≥8 h/day [20]. Both exposures were self-reported and measured once at baseline.

### Covariate selection and missing data

Covariates were selected a priori using a conceptual causal model and published evidence rather than automated significance testing [25]. In this model, age, sex, race/ethnicity, education, poverty-income ratio, smoking, alcohol use, and insurance coverage were common causes or proxies for common causes of activity patterns and mortality. CKM stage was included in the primary fully adjusted model to account for baseline CKM severity. The conceptual direction was: sociodemographic and access-to-care factors → LTPA/daily sitting time and mortality; smoking/alcohol → LTPA/daily sitting time and mortality; and CKM stage → activity limitations and mortality. This framework was used to avoid controlling indiscriminately for variables on the hypothesized pathway.

BMI, hypertension, and diabetes are components of CKM staging and may partly mediate associations between behavior and mortality; therefore, they were excluded from the primary confounder model and added only in a sensitivity model. A separate sensitivity model additionally included eGFR and log-transformed UACR. Smoking was classified as never, former, or current. Current alcohol use was coded “yes” for mild, moderate, or heavy current consumption and “no” for never or former consumption. Education, insurance, smoking, diabetes, and hypertension definitions are detailed in Tables S2–S4. Analyses used complete values for the prespecified primary covariates; no multiple imputation was applied.

### Outcomes

The primary outcome was all-cause mortality. Secondary outcomes were CVD and non-CVD mortality. Vital status and underlying cause of death were obtained from the National Death Index-linked public-use mortality files through December 31, 2019. CVD mortality included diseases of the heart (I00–I09, I11, I13, I20–I51) and cerebrovascular diseases (I60–I69); all other deaths were classified as non-CVD mortality [26].

### Statistical methodology

Analyses were performed in R 4.6.0 using the survey 4.5 and survival 3.8-6 packages. Because fasting glucose and triglycerides contributed to CKM staging, the 2-year fasting-subsample weight (WTSAF2YR) was divided by six to create a 12-year weight for 2007–2018. Survey objects specified strata (SDMVSTRA), primary sampling units (SDMVPSU), the 12-year fasting weight, and nesting. Participants with WTSAF2YR = 0 were excluded. Weighted estimates therefore target US noninstitutionalized adults represented by the fasting subsample who met the analytic eligibility and complete-case criteria; the complete-case restriction means full national representativeness cannot be guaranteed.

Continuous variables are presented as survey-weighted means with standard errors, and categorical variables as unweighted counts with survey-weighted percentages. Group differences were evaluated with design-adjusted Wald tests or Rao–Scott tests. Survey-weighted Kaplan–Meier curves and survey-adjusted log-rank tests were used for descriptive survival comparisons. For each joint exposure group, unweighted events, survey-weighted person-years, and mortality rates per 1,000 person-years with 95% CIs were calculated.

For joint analyses, LTPA was dichotomized as sufficient (≥150 min/week) versus <150 min/week (inactive or insufficient) and crossed with the three sitting-time categories to form six groups; sufficient LTPA with sitting <6 h/day was the reference. This six-level variable estimates joint-category associations but is not itself an interaction test. For formal interaction analyses, LTPA was dichotomized as <150 versus ≥150 min/week and sitting time as ≥8 versus <8 h/day. Multiplicative interaction was assessed with the single LTPA-by-sitting product term in the survey Cox model. RERI, attributable proportion, and the synergy index were derived from the same model coefficients with delta-method 95% CIs. Thus, multiplicative and additive interaction used the same 2 × 2 exposure definition. Sitting-time contrasts were also estimated within LTPA strata and LTPA contrasts within sitting-time strata.

Survey-design-based Cox proportional hazards models estimated HRs and 95% CIs. Model 1 was unadjusted. Model 2 adjusted for age, sex, and race/ethnicity. Model 3 additionally adjusted for education, poverty-income ratio, smoking, alcohol use, insurance, and CKM stage. BMI, hypertension, and diabetes were added in a mediator-adjusted sensitivity model; eGFR and UACR were added in a separate renal sensitivity model. All-cause mortality was analyzed as the primary endpoint; cause-specific Cox models treated deaths from other causes as censoring.

Proportional hazards were evaluated with scaled Schoenfeld-residual tests. Model discrimination was summarized with Harrell concordance; global design-adjusted Wald tests assessed overall model significance. Prespecified primary sensitivity analyses: (1) restricted age to 30–79 years; (2) restricted CKM to stages 0–2; (3) required complete BMI, waist, glucose, HbA1c, triglyceride, HDL, eGFR, and UACR data; (4) excluded deaths within two years; (5) required ≥2 years of follow-up; (6) added BMI, hypertension, and diabetes; and (7) added eGFR and log(UACR + 1). Supplemental analyses restricted age to ≥45 years, excluded participants with baseline cancer, and applied survey-weighted Fine–Gray competing-risk models to cause-specific outcomes. Effect modification by age group and sex was tested with design-adjusted Wald tests. For CKM-stratum analyses, stages 0 and 1 were pooled because separate early-stage cause-specific cells were sparse. A single survey Cox model directly compared the two prespecified extreme categories—<150 min/week with sitting ≥8 h/day versus sufficient LTPA with sitting <6 h/day—using shared covariate slopes and exposure-by-CKM-stratum terms. Tests were two-sided with P < 0.05.

## Results

The survey-weighted mean age was 51.31 years (SE, 0.22); 52.16% were female and 70.15% were non-Hispanic White. CKM stages 0, 1, 2, and 3 included 494, 1,667, 5,667, and 728 participants, respectively. Baseline characteristics by daily sitting time are shown in Table 1. Sitting-time categories differed with respect to age, race/ethnicity, education, poverty-income ratio, BMI, smoking, current alcohol use, insurance, and hypertension, but not sex, diabetes, CKM stage, or LTPA. Baseline characteristics by LTPA are shown in Table S5. Participants with sufficient LTPA were younger; more often male, non-Hispanic White, college educated, in the highest poverty-income-ratio category, never-smokers, current alcohol users, and privately insured; and had lower BMI and lower prevalences of hypertension, diabetes, and stage 3 CKM. Daily sitting-time distribution did not differ across LTPA categories (P = 0.158). Daily sitting time and LTPA were weakly and non-significantly correlated (survey-weighted r = −0.020; design-adjusted P = 0.134; Fig. S2).

**Table 1:** Characteristics of the primary analytic cohort by daily sitting time.

**Characteristic**	**Total**	**<6 h/day**	**6 to <8 h/day**	**≥8 h/day**	**P value**
**Age, mean (SE), years**	51.31(0.22)	51.17(0.29)	52.49(0.49)	50.99(0.33)	.026
**Sex, n (weighted %)**					.441
Female	4440(52.16)	2371(53.11)	662(52.05)	1407(50.97)	
Male	4116(47.84)	2139(46.89)	638(47.95)	1339(49.03)	
**Race/ethnicity, n (weighted %)**					<0.001
Mexican American	1290(7.63)	888(10.82)	159(6.04)	243(4.19)	
Non-Hispanic Black	1627(10.08)	822(10.42)	252(9.97)	553(9.68)	
Non-Hispanic White	3760(70.15)	1740(64.18)	627(73.59)	1393(76.42)	
Other Race	1879(12.14)	1060(14.59)	262(10.40)	557(9.71)	
**Education, n (weighted %)**					<0.001
<High school	1984(14.79)	1337(19.61)	261(13.31)	386(9.18)	
High school	1905(22.05)	1072(25.13)	297(22.90)	536(17.71)	
College or above	4667(63.16)	2101(55.26)	742(63.80)	1824(73.12)	
**PIR, n (weighted %)**					<0.001
<1.3	2449(18.12)	1503(22.56)	347(15.66)	599(13.44)	
1.3–<3.5	3211(34.88)	1815(39.51)	478(35.37)	918(28.69)	
>=3.5	2896(46.99)	1192(37.93)	475(48.97)	1229(57.87)	
**BMI, mean (SE), kg/m2**	29.38(0.12)	28.71(0.16)	29.66(0.31)	30.14(0.19)	<0.001
**Smoking status, n (weighted %)**					<0.001
Never	4717(55.41)	2529(54.59)	696(53.81)	1492(57.14)	
Former	2233(26.90)	1109(25.15)	354(28.76)	770(28.36)	
Current	1606(17.70)	872(20.27)	250(17.43)	484(14.50)	
**Current alcohol use, n (weighted %)**					<0.001
No	2741(25.52)	1529(27.85)	426(26.93)	786(21.91)	
Yes	5815(74.48)	2981(72.15)	874(73.07)	1960(78.09)	
**Insurance, n (weighted %)**					<0.001
Private	3801(56.47)	1756(50.67)	589(55.98)	1456(64.17)	
Medicare	1972(18.51)	1006(18.56)	355(22.45)	611(16.78)	
Medicaid	497(4.09)	288(4.90)	70(3.41)	139(3.34)	
Other	178(1.60)	107(1.90)	23(1.22)	48(1.38)	
None	2108(19.33)	1353(23.98)	263(16.94)	492(14.33)	
**Hypertension, n (weighted %)**					.036
No	4654(58.36)	2551(60.32)	688(57.50)	1415(56.19)	
Yes	3902(41.64)	1959(39.68)	612(42.50)	1331(43.81)	
**Diabetes mellitus, n (weighted %)**					.157
No	6644(83.13)	3532(84.30)	1004(81.95)	2108(82.11)	
Yes	1912(16.87)	978(15.70)	296(18.05)	638(17.89)	
**CKM stage, n (weighted %)**					.103
Stage 0	494(7.41)	269(8.12)	70(6.45)	155(6.91)	
Stage 1	1667(22.48)	891(21.96)	242(22.06)	534(23.33)	
Stage 2	5667(64.90)	3025(65.64)	859(65.38)	1783(63.75)	
Stage 3	728(5.21)	325(4.28)	129(6.11)	274(6.01)	
**LTPA, n (weighted %)**					.158
Inactive	4536(47.13)	2429(47.64)	698(50.05)	1409(45.22)	
Insufficient	1360(17.55)	673(16.74)	210(16.13)	477(19.20)	
Sufficient	2660(35.33)	1408(35.63)	392(33.82)	860(35.58)	

During a median follow-up of 6.75 years, 670 deaths occurred, including 175 CVD and 495 non-CVD deaths. Survey-weighted Kaplan–Meier curves differed across LTPA categories for all-cause, CVD, and non-CVD mortality (all survey-adjusted log-rank P < 0.001; Fig. S3). In Model 3, compared with inactive participants, sufficient LTPA was associated with lower all-cause (hazard ratio [HR], 0.630; 95% confidence interval [CI], 0.483–0.822; P < 0.001), CVD (HR, 0.536; 95% CI, 0.297–0.969; P = 0.039), and non-CVD mortality (HR, 0.662; 95% CI, 0.478–0.916; P = 0.013). Insufficient LTPA was also associated with all-cause (HR, 0.593; 95% CI, 0.422–0.834; P = 0.003), CVD (HR, 0.493; 95% CI, 0.248–0.981; P = 0.044), and non-CVD mortality (HR, 0.626; 95% CI, 0.424–0.925; P = 0.019). Per additional 60 moderate-equivalent min/week, HRs were 0.952 (95% CI, 0.919–0.986; P = 0.007), 0.895 (95% CI, 0.821–0.977; P = 0.013), and 0.965 (95% CI, 0.929–1.002; P = 0.062), respectively (Table 2). When grouped by daily sitting time, survival differed for all-cause mortality (P = 0.023), but not for CVD (P = 0.076) or non-CVD mortality (P = 0.168; Fig. S4). In Model 3, compared with sitting <6 h/day, sitting ≥8 h/day was associated with all-cause (HR, 1.506; 95% CI, 1.253–1.810; P < 0.001), CVD (HR, 1.845; 95% CI, 1.246–2.732; P = 0.002), and non-CVD mortality (HR, 1.406; 95% CI, 1.109–1.782; P = 0.005) (Table 2).

**Table 2:** Independent associations of LTPA and daily sitting time with mortality.

**Exposure / outcome**	**Events, ** **n**	**Model 1 HR** **(95% CI)**	**P**	**Model 2 HR** **(95% CI)**	**P**	**Model 3 HR** **(95% CI)**	**P**
**Leisure-time physical activity**							
All-cause mortality: Per additional 60 min/week	670	0.884 (0.843–0.926)	<0.001	0.917 (0.878–0.956)	<0.001	0.952 (0.919–0.986)	.007
CVD mortality: Per additional 60 min/week	175	0.804 (0.723–0.894)	<0.001	0.848 (0.770–0.933)	<0.001	0.895 (0.821–0.977)	.013
Non-CVD mortality: Per additional 60 min/week	495	0.903 (0.859–0.950)	<0.001	0.933 (0.891–0.977)	.003	0.965 (0.929–1.002)	.062
**Leisure-time physical activity categories**							
All-cause mortality — Inactive (0 min/week)	482	1.000 (1.000–1.000)		1.000 (1.000–1.000)		1.000 (1.000–1.000)	
Insufficient (>0 to <150 min/week)	69	0.376 (0.271–0.524)	<0.001	0.457 (0.328–0.637)	<0.001	0.593 (0.422–0.834)	.003
Sufficient (>=150 min/week)	119	0.342 (0.267–0.437)	<0.001	0.449 (0.348–0.579)	<0.001	0.630 (0.483–0.822)	<0.001
CVD mortality — Inactive (0 min/week)	129	1.000 (1.000–1.000)		1.000 (1.000–1.000)		1.000 (1.000–1.000)	
Insufficient (>0 to <150 min/week)	17	0.296 (0.154–0.571)	<0.001	0.378 (0.196–0.728)	.004	0.493 (0.248–0.981)	.044
Sufficient (>=150 min/week)	29	0.270 (0.163–0.446)	<0.001	0.370 (0.217–0.630)	<0.001	0.536 (0.297–0.969)	.039
Non-CVD mortality — Inactive (0 min/week)	353	1.000 (1.000–1.000)		1.000 (1.000–1.000)		1.000 (1.000–1.000)	
Insufficient (>0 to <150 min/week)	52	0.407 (0.280–0.591)	<0.001	0.486 (0.334–0.707)	<0.001	0.626 (0.424–0.925)	.019
Sufficient (>=150 min/week)	90	0.369 (0.272–0.502)	<0.001	0.478 (0.350–0.652)	<0.001	0.662 (0.478–0.916)	.013
**Daily sitting time**							
All-cause mortality: Per additional hour/day	670	1.037 (1.014–1.061)	.002	1.050 (1.022–1.078)	<0.001	1.060 (1.036–1.084)	<0.001
CVD mortality: Per additional hour/day	175	1.067 (1.018–1.118)	.007	1.089 (1.031–1.150)	.002	1.108 (1.050–1.168)	<0.001
Non-CVD mortality: Per additional hour/day	495	1.027 (0.997–1.058)	.080	1.037 (1.000–1.074)	.047	1.044 (1.013–1.077)	.005
**Daily sitting time categories**							
All-cause mortality — <6 h/day	300	1.000 (1.000–1.000)		1.000 (1.000–1.000)		1.000 (1.000–1.000)	
6 to <8 h/day	112	1.181 (0.842–1.657)	.334	1.014 (0.734–1.400)	.934	0.983 (0.722–1.338)	.912
>=8 h/day	258	1.345 (1.090–1.660)	.006	1.426 (1.145–1.776)	.002	1.506 (1.253–1.810)	<0.001
CVD mortality — <6 h/day	72	1.000 (1.000–1.000)		1.000 (1.000–1.000)		1.000 (1.000–1.000)	
6 to <8 h/day	31	1.034 (0.583–1.835)	.908	0.857 (0.493–1.490)	.584	0.863 (0.504–1.478)	.592
>=8 h/day	72	1.556 (1.038–2.332)	.032	1.665 (1.103–2.513)	.015	1.845 (1.246–2.732)	.002
Non-CVD mortality — <6 h/day	228	1.000 (1.000–1.000)		1.000 (1.000–1.000)		1.000 (1.000–1.000)	
6 to <8 h/day	81	1.229 (0.846–1.786)	.278	1.065 (0.742–1.529)	.732	1.023 (0.716–1.461)	.900
>=8 h/day	186	1.276 (0.987–1.650)	.063	1.346 (1.026–1.766)	.032	1.406 (1.109–1.782)	.005

Model 1: unadjusted. Model 2: age, sex, and race/ethnicity. Model 3: Model 2 plus education, poverty-income ratio, smoking, current alcohol use, insurance, and CKM stage. HRs use survey-design-based Cox models. The LTPA continuous estimate is per additional 60 moderate-equivalent min/week.

In the joint analysis of LTPA and sitting time, survey-weighted survival probabilities differed across the six groups for all-cause, CVD, and non-CVD mortality (all survey-adjusted log-rank P < 0.001), with the lowest survival generally observed among participants with <150 min/week of LTPA and sitting ≥8 h/day (Fig. 1). Compared with sufficient LTPA and sitting <6 h/day, this least favorable joint category was associated with all-cause (HR, 1.972; 95% CI, 1.429–2.721; P < 0.001), CVD (HR, 2.493; 95% CI, 1.232–5.045; P = 0.011), and non-CVD mortality (HR, 1.806; 95% CI, 1.202–2.712; P = 0.004) (Table 3; Fig. 2). Corresponding survey-weighted mortality rates were 13.13 versus 4.43 all-cause, 4.23 versus 1.11 CVD, and 8.89 versus 3.32 non-CVD deaths per 1,000 person-years. Within the <150-min/week stratum, sitting ≥8 versus <6 h/day was associated with all-cause (HR, 1.487; 95% CI, 1.206–1.834; P < 0.001), CVD (HR, 2.051; 95% CI, 1.319–3.188; P = 0.001), and non-CVD mortality (HR, 1.315; 95% CI, 1.018–1.698; P = 0.036). Within the sufficient-LTPA stratum, the corresponding estimates were imprecise and not statistically significant (HRs, 1.443, 0.675, and 1.682, respectively; Fig. S5).

**Fig. 1:**
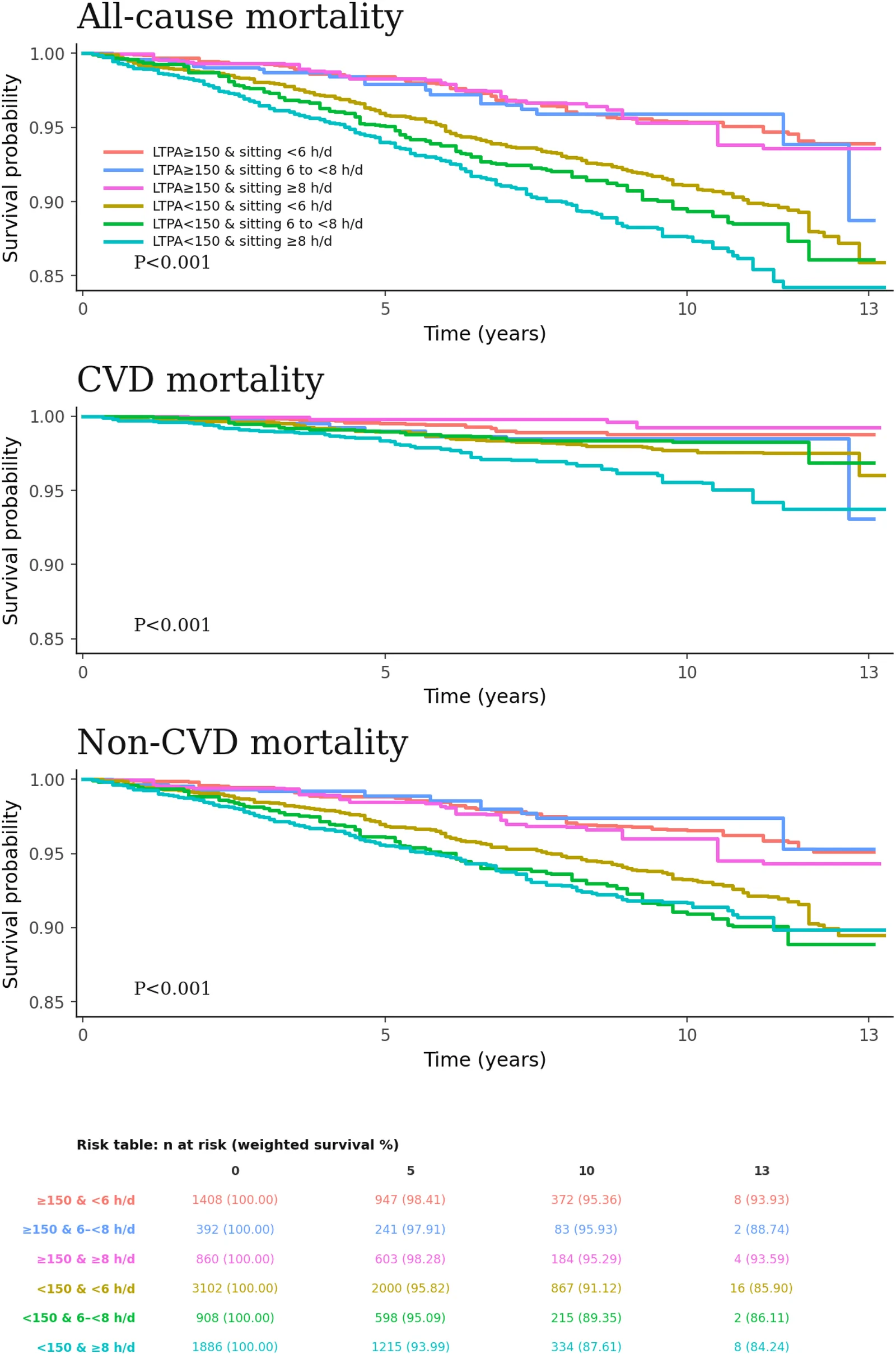
Survey-weighted Kaplan–Meier survival by joint LTPA and sitting categories. Abbreviations: LTPA, leisure-time physical activity; CVD, cardiovascular disease. Sufficient LTPA: ≥150 min/week; less than sufficient LTPA: <150 min/week. Survey-adjusted log-rank P < 0.001 for each outcome. Numbers at risk are unweighted participant counts.

**Table 3:** Joint associations, weighted person-years, and mortality rates.

**Joint LTPA / sitting group**	**n / events**	**Weighted PY (million) / rate per 1,000 PY**	**Model 1 HR (95% CI)**	**P**	**Model 2 HR (95% CI)**	**P**	**Model 3 HR (95% CI)**	**P**
**All-cause mortality**								
Sufficient & <6 h/d	1408 / 65	165.5 / 4.43 (3.06–5.80)	1.000 (1.000–1.000)		1.000 (1.000–1.000)		1.000 (1.000–1.000)	
Sufficient & 6–<8 h/d	392 / 19	46.4 / 4.49 (2.17–6.81)	1.038 (0.546–1.974)	.910	0.860 (0.484–1.528)	.607	0.833 (0.479–1.450)	.519
Sufficient & >=8 h/d	860 / 35	124.4 / 4.44 (2.53–6.36)	1.009 (0.571–1.783)	.975	1.240 (0.712–2.159)	.447	1.443 (0.853–2.441)	.172
<150 min/wk & <6 h/d	3102 / 235	296.4 / 9.16 (7.57–10.74)	2.063 (1.450–2.935)	<0.001	1.758 (1.249–2.474)	.001	1.326 (0.951–1.850)	.096
<150 min/wk & 6–<8 h/d	908 / 93	99.1 / 10.68 (7.33–14.03)	2.425 (1.484–3.961)	<0.001	1.818 (1.166–2.835)	.008	1.346 (0.868–2.086)	.184
<150 min/wk & >=8 h/d	1886 / 223	208.6 / 13.13 (11.04–15.22)	3.022 (2.142–4.263)	<0.001	2.514 (1.821–3.472)	<0.001	1.972 (1.429–2.721)	<0.001
**CVD mortality**								
Sufficient & <6 h/d	1408 / 15	165.5 / 1.11 (0.44–1.78)	1.000 (1.000–1.000)		1.000 (1.000–1.000)		1.000 (1.000–1.000)	
Sufficient & 6–<8 h/d	392 / 8	46.4 / 1.76 (0.41–3.11)	1.613 (0.628–4.147)	.321	1.234 (0.520–2.929)	.633	1.258 (0.531–2.981)	.603
Sufficient & >=8 h/d	860 / 6	124.4 / 0.47 (0.06–0.87)	0.418 (0.140–1.247)	.118	0.550 (0.184–1.647)	.286	0.675 (0.242–1.878)	.451
<150 min/wk & <6 h/d	3102 / 57	296.4 / 2.23 (1.39–3.08)	2.006 (0.957–4.203)	.065	1.680 (0.796–3.546)	.174	1.216 (0.552–2.679)	.628
<150 min/wk & 6–<8 h/d	908 / 23	99.1 / 1.93 (0.88–2.98)	1.746 (0.754–4.040)	.193	1.271 (0.555–2.911)	.571	0.933 (0.384–2.266)	.878
<150 min/wk & >=8 h/d	1886 / 66	208.6 / 4.23 (3.10–5.36)	3.867 (1.949–7.673)	<0.001	3.114 (1.578–6.147)	.001	2.493 (1.232–5.045)	.011
**Non-CVD mortality**								
Sufficient & <6 h/d	1408 / 50	165.5 / 3.32 (2.06–4.59)	1.000 (1.000–1.000)		1.000 (1.000–1.000)		1.000 (1.000–1.000)	
Sufficient & 6–<8 h/d	392 / 11	46.4 / 2.73 (0.90–4.56)	0.844 (0.372–1.912)	.684	0.716 (0.327–1.568)	.403	0.689 (0.322–1.477)	.338
Sufficient & >=8 h/d	860 / 29	124.4 / 3.98 (2.04–5.92)	1.210 (0.624–2.347)	.573	1.458 (0.759–2.801)	.258	1.682 (0.894–3.165)	.107
<150 min/wk & <6 h/d	3102 / 178	296.4 / 6.92 (5.59–8.25)	2.082 (1.332–3.254)	.001	1.788 (1.152–2.776)	.010	1.373 (0.907–2.079)	.134
<150 min/wk & 6–<8 h/d	908 / 70	99.1 / 8.75 (5.78–11.73)	2.651 (1.502–4.679)	<0.001	2.009 (1.188–3.399)	.009	1.498 (0.899–2.496)	.121
<150 min/wk & >=8 h/d	1886 / 157	208.6 / 8.89 (7.34–10.45)	2.737 (1.789–4.187)	<0.001	2.300 (1.527–3.464)	<0.001	1.806 (1.202–2.712)	.004

PY, person-years. Model definitions are given in Table 2. The reference is sufficient LTPA with sitting <6 h/day. Counts are unweighted; person-years and rates are survey weighted.

**Fig. 2:**
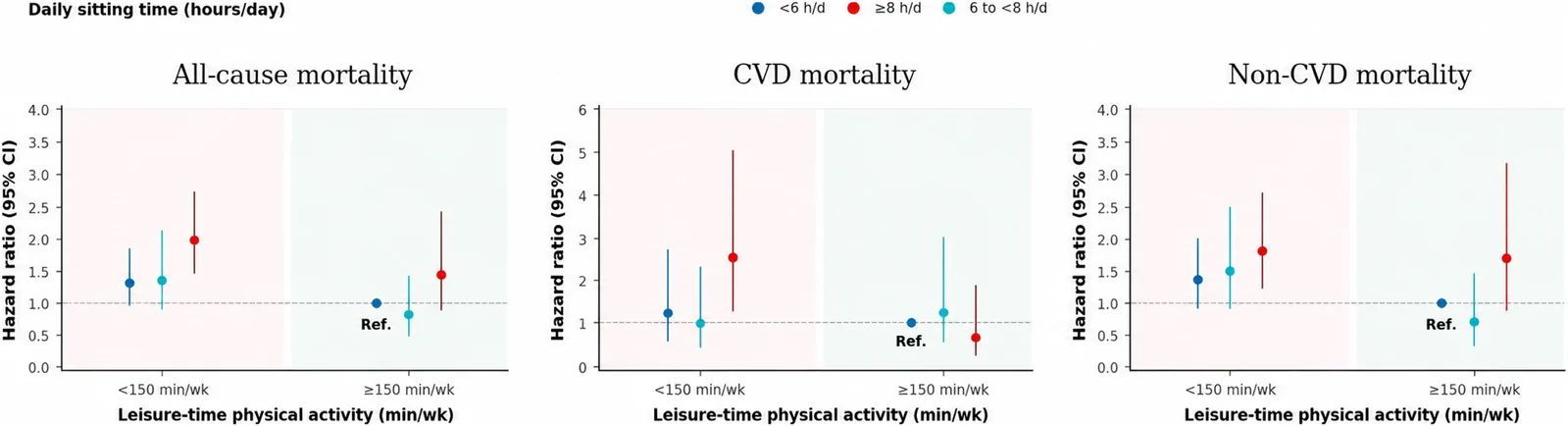
Joint associations of LTPA and sitting time with mortality in CKM stages 0–3. Abbreviations: LTPA, leisure-time physical activity; CKM, cardiovascular-kidney-metabolic; CVD, cardiovascular disease; HR, hazard ratio. Survey-design-based Cox models adjusted for age, sex, race/ethnicity, education, poverty-income ratio, smoking, current alcohol use, insurance, and CKM stage. Sufficient LTPA with sitting <6 h/day was the reference for the displayed six-group estimates. Formal multiplicative interaction used LTPA <150 versus ≥150 min/week and sitting ≥8 versus <8 h/day.

The associations for the least favorable joint category remained directionally similar in the prespecified analyses reported in Table 4 and in supplemental analyses. After excluding deaths within two years, HRs were 1.887 (95% CI, 1.349–2.640) for all-cause, 2.724 (95% CI, 1.319–5.627) for CVD, and 1.640 (95% CI, 1.093–2.462) for non-CVD mortality (Fig. S6); after requiring at least two years of follow-up, the corresponding HRs were 1.907 (95% CI, 1.374–2.646), 2.634 (95% CI, 1.307–5.307), and 1.682 (95% CI, 1.123–2.519) (Fig. S7). Estimates were also directionally consistent among participants aged ≥45 years (HRs, 2.198, 3.215, and 1.915; Fig. S8), among those with complete CKM laboratory data (HRs, 1.922, 2.810, and 1.689; Fig. S9), and after excluding baseline cancer (HRs, 3.023, 2.411, and 3.715; Fig. S10). In survey-weighted Fine–Gray analyses, the corresponding subdistribution HRs were 2.440 (95% CI, 1.229–4.842; P = 0.011) for CVD and 1.699 (95% CI, 1.125–2.564; P = 0.012) for non-CVD mortality (Fig. S11). No effect modification was detected by age group (P = 0.505, 0.318, and 0.583 for all-cause, CVD, and non-CVD mortality; Fig. S12) or sex (P = 0.643, 0.475, and 0.753; Fig. S13).

**Table 4:** Sensitivity analyses for <150 min/week LTPA with sitting ≥8 h/day.

**Analysis (n)**	**All-cause HR (95% CI)**	**CVD HR (95% CI)**	**Non-CVD HR (95% CI)**
Primary (n = 8,556)	1.972 (1.429–2.721)	2.493 (1.232–5.045)	1.806 (1.202–2.712)
PREVENT age 30–79 (n = 8,139)	1.986 (1.361–2.897)	3.816 (1.600–9.102)	1.622 (1.042–2.526)
CKM stages 0–2 only (n = 7,828)	1.851 (1.224–2.799)	3.720 (1.532–9.033)	1.476 (0.898–2.425)
Complete CKM laboratory data (n = 8,210)	1.922 (1.386–2.665)	2.810 (1.394–5.665)	1.689 (1.123–2.538)
Exclude deaths within 2 years (n = 8,424)	1.887 (1.349–2.640)	2.724 (1.319–5.627)	1.640 (1.093–2.462)
Follow-up ≥2 years (n = 7,813)	1.907 (1.374–2.646)	2.634 (1.307–5.307)	1.682 (1.123–2.519)
Add BMI, hypertension, and diabetes (n = 8,493)	1.945 (1.421–2.661)	2.203 (1.092–4.444)	1.860 (1.249–2.768)
Add eGFR and log(UACR + 1) (n = 8,393)	1.774 (1.282–2.455)	2.174 (1.058–4.465)	1.644 (1.092–2.474)

Reference: sufficient LTPA with sitting <6 h/day. Unless otherwise specified, covariates followed Model 3.

Formal interaction results are shown in Table S6. Using the same 2 × 2 exposure definition for both scales, multiplicative interaction was not detected for all-cause (P = 0.951) or non-CVD mortality (P = 0.274), whereas the CVD interaction test was P = 0.016. Additive interaction was not detected for all-cause or non-CVD mortality. For CVD mortality, RERI was 1.641 (95% CI, 0.765–2.518) and the attributable proportion was 0.701 (95% CI, 0.370–1.032); the conventional synergy index was not reported because its denominator was non-positive. In the stabilized CKM-stratum analysis, the all-cause HR for the least favorable versus reference category was 1.933 (95% CI, 0.787–4.750) in pooled stages 0–1, 1.819 (95% CI, 1.144–2.893) in stage 2, and 2.059 (95% CI, 1.070–3.960) in stage 3 (P for heterogeneity = 0.958). The corresponding CVD HRs were 2.379, 3.694, and 1.308 (P for heterogeneity = 0.402), and non-CVD HRs were 1.770, 1.451, and 2.862 (P for heterogeneity = 0.275) (Table S7). Proportional-hazards tests showed no violation for the joint exposure (P = 0.844, 0.852, and 0.603 for all-cause, CVD, and non-CVD mortality; global P = 0.310, 0.942, and 0.199). Concordance statistics were 0.843, 0.897, and 0.829, and global design-adjusted Wald tests were all P < 0.001. Taken together, the joint categories identified a graded, higher-risk behavioral phenotype; however, the absence of multiplicative and additive interaction for all-cause and non-CVD mortality and the instability of the CVD interaction signal do not establish that sufficient LTPA offsets the mortality risk associated with prolonged sitting.

## Discussion

In this survey-weighted cohort of adults classified as CKM stages 0–3, higher LTPA and shorter daily sitting time were independently associated with lower mortality. Adults with <150 min/week of LTPA and sitting ≥8 h/day had the highest adjusted mortality and absolute rates relative to the sufficient-LTPA/<6-h reference. However, formal multiplicative interaction was absent for the primary all-cause outcome and for non-CVD mortality. The CVD interaction signal arose in the setting of sparse joint-category events and should be considered exploratory. Thus, the combined categories aid risk description but do not demonstrate that one behavior modifies or offsets the effect of the other.

LTPA is a cornerstone of metabolic and cardiovascular health and has been consistently associated with lower risks of CVD and all-cause mortality [27, 28]. Sufficient LTPA may also reduce the risk of metabolic disorders, CKD, and CVD [9, 29]. Notably, compared with complete leisure-time inactivity, even activity levels below the recommended minimum have been associated with an approximately 20% reduction in all-cause mortality risk, with further reductions observed at higher activity levels across age, sex, and racial groups [30]. Conversely, sedentary behavior is highly prevalent among adults and has been independently associated with cardiovascular events, kidney dysfunction, metabolic abnormalities, and premature mortality [31–33]. These findings suggest that LTPA and daily sitting time may provide complementary information for lifestyle-related risk assessment, particularly during CKM stages preceding clinically manifest CVD.

In the present study, when LTPA and sitting time were examined separately, adults with CKM stages 0–3 who reported no LTPA or daily sitting time ≥8 hours had higher mortality risks after adjustment for demographic, socioeconomic, lifestyle, and clinical covariates. The continuous analyses provided broadly consistent findings: greater moderate-equivalent LTPA was associated with lower all-cause and CVD mortality, whereas longer sitting time was associated with higher risk. These observations are consistent with previous cohort studies. Diaz et al., in an analysis of 7,985 US adults aged ≥45 years, reported that prolonged sedentary time was associated with increased all-cause mortality [34]. Similarly, among 1,554 adults with CKD, Ricardo et al. found that meeting physical-activity recommendations was associated with a substantially lower mortality risk [35]. However, much of the previous evidence has focused on individual CKM components, selected clinical populations, or either physical activity or sedentary behavior considered in isolation.

Recently published CKM-specific studies examined physical activity or sedentary behavior in relation to CKM prevalence, CKM stage, or short-term mortality [15–17]. The present study extends this literature by incorporating prospective cause-specific mortality follow-up in a nationally representative US sample, simultaneous classification of LTPA and daily sitting time, survey-weighted absolute mortality rates, and formal multiplicative and additive interaction testing across CKM stages 0–3. These features represent an incremental contribution to the existing literature without implying that the present analysis is the first or definitive study of physical activity in CKM health.

Meta-analyses and large cohort studies in the general population have established that longer daily sitting time is associated with higher all-cause and cardiovascular mortality [11–13] and that leisure-time physical activity across the adult life course is associated with lower all-cause and cause-specific mortality [14]. Because adults with CKM stages 0–3 constitute a large proportion of the general population, the present findings are best understood as extending—rather than challenging—this evidence to a CKM-staging framework that links combined behavioral patterns to absolute and cause-specific mortality risk. The World Health Organization 2020 guidelines on physical activity and sedentary behavior and the second edition of the Physical Activity Guidelines for Americans recommend that adults undertake 150–300 minutes of moderate-intensity aerobic activity, 75–150 minutes of vigorous-intensity activity, or an equivalent combination each week, while also limiting sedentary time [24, 36]. Previous prospective studies have suggested that physical activity may modify the association between prolonged sitting and mortality [19, 37–39]. In a large cohort with a median follow-up of 8.9 years, the association of prolonged sitting with all-cause and cardiovascular mortality was most evident among individuals with the lowest activity levels; among those meeting activity recommendations, elevated risk was mainly observed in participants with very prolonged sitting who only achieved the minimum recommended activity level [19]. A harmonized meta-analysis similarly showed that the excess mortality associated with prolonged sitting was greatest among individuals in the lowest quartile of physical activity [39].

Our joint analyses provide a more cautious and CKM-specific interpretation of this relationship. Among participants performing <150 minutes/week of LTPA, sitting ≥8 hours/day was associated with higher mortality than sitting <6 hours/day. Among participants meeting the LTPA recommendation, however, estimates for different sitting-time categories were imprecise and their confidence intervals crossed unity. Moreover, the multiplicative interaction between LTPA and sitting time was not statistically significant for all-cause mortality. Therefore, although the joint-category results suggest that insufficient activity and prolonged sitting may identify a particularly unfavorable behavioral pattern, they do not establish that sufficient LTPA fully offsets the risk associated with prolonged sitting. Even without statistical interaction, the joint analysis is informative in three respects. First, it identified a sizeable subgroup—insufficiently active adults who sit ≥8 h/day—with an absolute all-cause mortality rate approximately three times that of the reference group (13.13 versus 4.43 deaths per 1,000 person-years), this subgroup comprised approximately one-fifth of the cohort (1,886 of 8,556 participants) yet accounted for one-third of all deaths (223 of 670), making it directly relevant to risk prioritization. Second, within-stratum contrasts showed that the sitting–mortality association was concentrated among insufficiently active adults, whereas estimates among sufficiently active adults were imprecise. Third, the absence of detectable interaction indicates that, at the population level, the two behaviors are associated with mortality largely independently and additively, so each warrants attention in its own right rather than one being expected to compensate for the other.

For CVD mortality, the significant multiplicative interaction and positive relative excess risk due to interaction suggest possible effect modification on multiplicative and additive scales. We interpret this signal as exploratory for several converging reasons rather than the event count alone. First, the signal depends on the sufficient-LTPA/≥8-h-sitting category, in which only six CVD deaths occurred and the within-stratum estimate for sitting ≥8 versus <6 h/day was below unity (HR, 0.675); the interaction therefore hinges on a protective point estimate supported by very few events, and reclassification of even a small number of deaths could materially change the P value. Second, the conventional synergy index was not reported because its mathematical denominator, (HR10−1) + (HR01−1), was non-positive—a mathematical limitation of the index rather than Cox-model non-convergence—indicating that the joint pattern departs from the standard form in which each exposure alone increases risk. Third, interaction was tested for three mortality outcomes, so a single P value of 0.016 should be weighed against multiplicity. Fourth, although the main joint association for CVD mortality remained directionally consistent across sensitivity analyses (excluding deaths within two years, requiring ≥2 years of follow-up, requiring complete CKM components, and excluding baseline cancer) and in survey-weighted Fine–Gray competing-risk models, and model fit was satisfactory (Schoenfeld P = 0.852; concordance 0.897), these analyses support the robustness of the main association rather than the stability of the interaction term itself. In the CKM-stage-stratified analyses, the least favorable joint behavioral pattern was associated with all-cause mortality in stages 2 and 3, but formal evidence of heterogeneity across pooled stages 0–1, stage 2, and stage 3 was not detected. Cause-specific estimates in the earlier CKM stages were also imprecise because of the limited number of events. Collectively, these results support consideration of both LTPA and sitting time but do not define a reliable CKM-specific activity threshold capable of compensating for prolonged sedentary behavior.

Several overlapping biological pathways could plausibly explain the independent and joint associations of LTPA and sedentary behavior with mortality. Prolonged sitting reduces skeletal-muscle contractile activity and may impair lipoprotein-lipase activity, postprandial glucose disposal, and lipid metabolism. It has also been associated with higher concentrations of inflammatory markers, including C-reactive protein and interleukin-6 [40–42]. Persistent systemic inflammation may interfere with insulin signaling, promote insulin resistance and dyslipidemia, and impair the anti-inflammatory and cholesterol-transport functions of lipoproteins [40, 41]. These abnormalities overlap closely with obesity, diabetes, hypertension, dyslipidemia, and kidney dysfunction, which are central components of CKM health.

Physical activity may exert potentially countervailing effects by improving insulin sensitivity, skeletal-muscle glucose uptake, mitochondrial function, vascular reactivity, and adipose-tissue metabolism. It may also reduce the production of proinflammatory cytokines and enhance anti-inflammatory signaling [43, 44]. In addition, oxidative stress and inflammation can reinforce each other, contributing to endothelial dysfunction, vascular injury, arterial remodeling, and progressive cardiac and kidney damage [45, 46]. Regular physical activity may enhance endogenous antioxidant capacity and reduce oxidative injury, whereas prolonged sedentary exposure may contribute to impaired redox balance and endothelial homeostasis [45, 47]. Importantly, physical activity and sedentary behavior are not exact physiological opposites. An individual may satisfy weekly activity recommendations while still accumulating long and uninterrupted periods of sitting; consequently, some adverse metabolic and vascular pathways related to sedentary exposure may persist despite otherwise adequate LTPA. Because inflammatory biomarkers, oxidative-stress markers, and repeated behavioral measurements were not comprehensively available in the present analysis, these mechanisms should be considered biologically plausible explanations rather than demonstrated mediators.

From a clinical perspective, LTPA and daily sitting time may provide complementary information when discussing lifestyle behaviors with adults across CKM stages 0–3. The lower mortality observed among participants with more favorable combinations of LTPA and sitting time is consistent with current recommendations to increase moderate-to-vigorous physical activity and reduce prolonged sedentary exposure. However, this study was observational and cannot establish that changing either behavior would reduce mortality by the magnitude estimated in the present models. The findings therefore support integrating assessment of both activity and sitting into comprehensive CKM lifestyle counseling, rather than using LTPA alone as a proxy for overall movement behavior. Randomized trials, quasi-experimental studies, and prospective cohorts using device-measured activity, sedentary duration, sitting-bout patterns, and repeated exposure assessments are needed to determine whether reducing sedentary time provides benefits beyond increasing LTPA and whether clinically meaningful compensation thresholds can be defined for adults at different CKM stages.

### Strengths and limitations

Strengths include use of six NHANES cycles with mortality linkage, fasting-subsample weights and design-based variance estimation, correction of cause-specific mortality to include heart and cerebrovascular deaths, reporting of absolute weighted rates, and formal multiplicative, additive, within-LTPA-stratum, and CKM-stratum analyses. Prespecified and supplemental sensitivity analyses addressed PREVENT age bounds, CKM stage, CKM-component completeness, early mortality, follow-up duration, baseline cancer, potential CKM-domain mediators, kidney markers, competing risks, and effect modification by age and sex.

Several limitations require emphasis. First, the observational design is subject to residual confounding and reverse causation and cannot establish an intervention effect. Second, LTPA and sitting time were self-reported once, creating recall error and exposure misclassification. Third, excluding 4,510 participants with missing covariates and 822 with zero fasting weights may have introduced selection bias; because the updated file did not retain excluded records, covariate-specific missing counts and a direct included-versus-excluded comparison could not be reconstructed, and national representativeness after complete-case restriction cannot be guaranteed. Fourth, CKM staging may be misclassified when component data were incomplete; PREVENT required age and variable truncation, and cardiac biomarkers and imaging were unavailable. Fifth, CVD deaths were sparse in several joint cells, including six deaths in the sufficient-LTPA/≥8-h group and only two reference versus four target events in pooled CKM stages 0–1, producing wide interaction and stratum-specific CIs. Sixth, HR-based additive-interaction indices approximate risk-scale interaction, and the conventional synergy index is undefined or not meaningful when its denominator is non-positive. Seventh, because CKM stages 0–3 encompass a large proportion of the general adult population, our cohort inevitably overlaps with the populations of previous general-population studies; the present analysis should therefore be viewed as extending that evidence to a CKM-staging framework rather than studying a fundamentally distinct population. Eighth, the analysis was prespecified primarily around main-effect and joint-category estimation; with only 175 CVD deaths, power to detect modest multiplicative or additive interactions was limited, and several stage-stratified cells—particularly pooled stages 0–1 and stage 3—were small. The absence of detected interaction should therefore not be interpreted as proof of no interaction, and larger cohorts with more cause-specific events will be needed to definitively resolve whether LTPA modifies the association between prolonged sitting and mortality. Finally, results apply to US noninstitutionalized adults aged ≥30 years represented by the fasting subsample and may not generalize to younger adults, stage 4, or other populations.

## Conclusion

Among US adults classified as CKM stages 0–3, higher LTPA and shorter daily sitting time were independently associated with lower all-cause and cause-specific mortality, and the combination of <150 min/week of LTPA with sitting ≥8 h/day identified a higher-risk group. Because interaction was not detected for the primary outcome and the study was observational, these findings do not show that LTPA eliminates the risk of prolonged sitting; intervention studies are needed to determine causal, CKM-specific behavioral targets.

## Supplementary information

**Additional file 1:** **Table S1.** Definition of CKM Syndrome stages 0–3. **Table S2.** Definition of smoking status. **Table S3.** Definition of insurance. **Table S4.** Definition of diabetes mellitus and hypertension. **Table S5.** Characteristics of the primary analytic cohort according to leisure-time physical activity. **Table S6.** Formal multiplicative and additive interaction analyses. **Table S7.** CKM-stratum-specific contrast between the least favorable and reference joint categories. **Figure S1.** Flow chart for participant selection. **Figure S2.** Relation between daily sitting time and leisure-time physical activity in the primary analytic cohort. **Figure S3.** Survey-weighted Kaplan-Meier curves for all-cause, CVD, and non-CVD mortality, stratified by leisure-time physical activity. **Figure S4.** Survey-weighted Kaplan-Meier curves for all-cause, CVD, and non-CVD mortality, stratified by daily sitting time. **Figure S5.** Associations of daily sitting time with mortality among US adults classified as CKM stages 0–3, stratified by leisure-time physical activity. **Figure S6.** Joint associations of leisure-time physical activity and daily sitting time with mortality after excluding deaths within 2 years. **Figure S7.** Joint associations of leisure-time physical activity and daily sitting time with mortality among participants followed for at least 2 years. **Figure S8.** Joint associations of leisure-time physical activity and daily sitting time with mortality among participants aged at least 45 years. **Figure S9.** Joint associations of leisure-time physical activity and daily sitting time with mortality among participants with complete CKM laboratory component data. **Figure S10.** Joint associations of leisure-time physical activity and daily sitting time with mortality after excluding participants with baseline cancer. **Figure S11.** Joint associations of leisure-time physical activity and daily sitting time with CVD and non-CVD mortality in weighted subdistribution hazard models. **Figure S12.** Joint associations of leisure-time physical activity and daily sitting time with mortality, stratified by age. **Figure S13.** Joint associations of leisure-time physical activity and daily sitting time with mortality, stratified by sex.

## Declarations

### Data availability statement

All data are available as publicly accessible datasets through NHANES. It is open and publicly accessible through the following link; https://wwwn.cdc.gov/nchs/nhanes/.
