# Supplementary material for: Joint associations of leisure-time physical activity and daily sitting time with mortality among US adults classified as CKM stages 0–3: a prospective cohort study of NHANES 2007–2018
Source: Environ Health Prev Med. 2026 Oct 20;31:69. doi: 10.1265/ehpm.26-00088 (PMC13719245; doi:10.1265/ehpm.26-00088)
Supplement: Supplementary file 1 [file ehpm-31-069-s001.pdf]

SUPPLEMENTAL CONTENT

Table S1. Definition of CKM Syndrome stages 0–3.
Table S2. Definition of smoking status.
Table S3. Definition of insurance.
Table S4. Definition of diabetes mellitus and hypertension.
Table S5. Characteristics of the primary analytic cohort according to leisure-time physical activity.
Table S6. Formal multiplicative and additive interaction analyses.
Table S7. CKM-stratum-specific contrast between the least favorable and reference joint categories.
Figure S1. Flow chart for participant selection.
Figure S2. Relation between daily sitting time and leisure-time physical activity.
Figure S3. Survey-weighted Kaplan–Meier curves stratified by leisure-time physical activity.
Figure S4. Survey-weighted Kaplan–Meier curves stratified by daily sitting time.
Figure S5. Sitting-time associations stratified by leisure-time physical activity.
Figures S6–S11. Prespecified sensitivity and competing-risk analyses.
Figure S12. Joint associations stratified by age.
Figure S13. Joint associations stratified by sex.

Table S1. Definition of CKM Syndrome stages 0–3.

CKM Stage 0	Participants with normal body mass index (BMI) (<23 kg/m2 for individuals with Asian ethnicity and <25 kg/m2 for other racial and ethnic groups), normal waist circumference (<80 and <90 cm for women and men with Asian race, respectively, and <88 and <102 cm for women and men in all other race and ethnicity categories, respectively) who did not meet criteria for the other stages.
CKM Stage 1	Participants with an elevated BMI (≥23 kg/m2 for individuals with Asian race and ≥25 kg/m2 for all other race and ethnic groups), an elevated waist circumference (≥80 and ≥90 cm for women and men with Asian race, respectively, and ≥88 and ≥102 cm for women and men in other race and ethnicity categories, respectively), or prediabetes (defined as a glycated hemoglobin of 5.7% to <6.5% or a fasting blood glucose of 100 mg/dL to <126 mg/dL).
CKM Stage 2	Participants with metabolic risk factors or moderate-to-high-risk CKD per Kidney Disease Improving Global Outcomes (KDIGO) criteria, as recommended by the AHA.1 Qualifying metabolic risk factors included elevated fasting serum triglycerides (≥135 mg/dL), hypertension, diabetes, or metabolic syndrome (≥3 of the following: elevated waist circumference, low high density lipoprotein cholesterol (HDL) level [<40 mg/dL or <50 mg/dL for men or women, respectively], fasting serum triglycerides ≥150 mg/dL, elevated blood pressure [systolic blood pressure ≥130, diastolic blood pressure ≥80 mmHg, and/or use of blood pressure-lowering medications], or prediabetes). CKD stages were identified based on eGFR and urinary albumin-to-creatinine ratio.
CKM Stage 3	Participants based of the presence of very-high-risk KDIGO CKD stages1 or a high-predicted 10-year CVD risk. 10-year cardiovascular risk was estimated with the AHA Predicting Risk of CVD EVENTs (PREVENT) equations. High risk was defined as ≥ 20% 10-year CVD risk (based on recommended thresholds [https://professional.heart.org/en/guidelines-and-statements/prevent-calculator]). The PREVENT equations were developed and validated for adults 30-79 years of age. As such, risk was not estimated for adults <30 years. However, to minimize underestimation of CKD Stage 3, adults ≥80 years were not excluded from 10-year CVD risk. Instead, adults ≥80 years were assigned an age of 79 years when determining 10-year CVD risk to allow for conservative estimates. Further, PREVENT was developed for variables with the following ranges: total cholesterol 130-320 mg/dL, HDL 20-100 mg/dL, systolic blood pressure 90-200 mmHg, and GFR 14-140 mL/min/1.73m^2 . To approximate PREVENT risk strata, values for these variables above or below these bounds were set to the upper or lower bounds of allowable values respectively (for example, total cholesterol of 330 mg/dL was set as 320 mg/dL). Cardiac biomarkers and cardiovascular imaging were not available to identify subclinical CVD.

Abbreviations: BMI, body mass index; CKD, chronic kidney disease; CKM, cardiovascular-kidney-metabolic; CVD, cardiovascular disease; eGFR, estimated glomerular filtration rate.

Table S2. Definition of smoking status.

**Current**	Smoked at least 100 cigarettes in the lifetime and currently smoked.
**Former**	Smoked at least 100 cigarettes in the lifetime but did not currently smoke.
**Never**	Smoked fewer than 100 cigarettes in the lifetime.

Table S3. Definition of insurance.

**Medicare**	Medicare, including Medicare together with another insurance type.
**Private**	Private insurance without Medicare.
**Medicaid**	Medicaid without Medicare or private insurance.
**Other**	Other government insurance without Medicare, private insurance, or Medicaid.
**None**	No insurance.

Table S4. Definition of diabetes mellitus and hypertension.

**Diabetes mellitus**	Any of: physician diagnosis; HbA1c ≥6.5%; fasting glucose ≥7.0 mmol/L; random glucose ≥11.1 mmol/L; 2-hour oral-glucose-tolerance-test glucose ≥11.1 mmol/L; or diabetes medication/insulin use.
**Hypertension**	Any of: self-reported diagnosis; measured systolic blood pressure ≥130 mmHg or diastolic blood pressure ≥80 mmHg; or current antihypertensive medication use.

Table S5. Characteristics of the primary analytic cohort according to leisure-time physical activity.

Characteristic	Total	Inactive (0 min/wk)	Insufficient (>0 to <150 min/wk)	Sufficient (≥150 min/wk)	P value
Age, mean (SE), years	51.31(0.22)	52.96(0.26)	51.25(0.45)	49.14(0.31)	<0.001
**Sex, n (weighted %)**					<0.001
Female	4440(52.16)	2426(53.68)	788(57.11)	1226(47.66)	
Male	4116(47.84)	2110(46.32)	572(42.89)	1434(52.34)	
**Race/ethnicity, n (weighted %)**					<0.001
Mexican American	1290(7.63)	779(9.35)	178(6.15)	333(6.07)	
Non-Hispanic Black	1627(10.08)	878(11.18)	248(8.59)	501(9.34)	
Non-Hispanic White	3760(70.15)	1925(67.10)	610(72.78)	1225(72.93)	
Other Race	1879(12.14)	954(12.37)	324(12.48)	601(11.66)	
**Education, n (weighted %)**					<0.001
<High school	1984(14.79)	1451(22.75)	209(9.88)	324(6.60)	
High school	1905(22.05)	1118(26.62)	288(20.48)	499(16.74)	
College or above	4667(63.16)	1967(50.64)	863(69.65)	1837(76.65)	
**PIR, n (weighted %)**					<0.001
<1.3	2449(18.12)	1618(25.21)	309(14.12)	522(10.66)	
1.3-<3.5	3211(34.88)	1801(39.42)	503(32.56)	907(29.98)	
>=3.5	2896(46.99)	1117(35.37)	548(53.32)	1231(59.36)	
BMI, mean (SE), kg/m2	29.38(0.12)	30.41(0.16)	29.34(0.22)	28.05(0.19)	<0.001
**Smoking status, n (weighted %)**					<0.001
Never	4717(55.41)	2335(50.96)	793(58.73)	1589(59.69)	
Former	2233(26.90)	1148(25.23)	373(28.10)	712(28.52)	
Current	1606(17.70)	1053(23.81)	194(13.17)	359(11.80)	
**Current alcohol use, n (weighted %)**					<0.001
No	2741(25.52)	1672(30.84)	402(23.83)	667(19.26)	
Yes	5815(74.48)	2864(69.16)	958(76.17)	1993(80.74)	
**Insurance, n (weighted %)**					<0.001
Private	3801(56.47)	1650(47.30)	688(60.46)	1463(66.71)	
Medicare	1972(18.51)	1197(21.62)	302(19.39)	473(13.93)	
Medicaid	497(4.09)	316(5.37)	62(3.21)	119(2.82)	
Other	178(1.60)	100(1.58)	23(1.46)	55(1.70)	
None	2108(19.33)	1273(24.13)	285(15.48)	550(14.84)	
**Hypertension, n (weighted %)**					<0.001
No	4654(58.36)	2274(52.56)	751(60.95)	1629(64.80)	
Yes	3902(41.64)	2262(47.44)	609(39.05)	1031(35.20)	
**Diabetes mellitus, n (weighted %)**					<0.001
No	6644(83.13)	3349(79.07)	1059(83.65)	2236(88.28)	
Yes	1912(16.87)	1187(20.93)	301(16.35)	424(11.72)	
**CKM stage, n (weighted %)**					<0.001
Stage 0	494(7.41)	183(4.73)	85(6.59)	226(11.40)	
Stage 1	1667(22.48)	748(18.91)	267(23.69)	652(26.64)	
Stage 2	5667(64.90)	3097(68.76)	918(65.93)	1652(59.24)	
Stage 3	728(5.21)	508(7.60)	90(3.78)	130(2.72)	
**Daily sitting time, n (weighted %)**					.158
<6 h/d	4510(47.55)	2429(48.07)	673(45.35)	1408(47.95)	
6-<8 h/d	1300(15.67)	698(16.64)	210(14.40)	392(15.00)	
>=8 h/d	2746(36.78)	1409(35.29)	477(40.25)	860(37.05)	

Table S6. Formal multiplicative and additive interaction analyses.

Outcome	Multiplicative interaction P	RERI (95% CI)	AP (95% CI)	Synergy index (95% CI)
All-cause mortality	.827	0.163 (-0.613–0.939)	0.079 (-0.299–0.457)	1.181 (0.150–2.212)
CVD mortality	.035	1.641 (0.765–2.518)	0.701 (0.370–1.032)	Not interpretable
Non-CVD mortality	.228	-0.393 (-1.470–0.684)	-0.201 (-0.739–0.338)	0.709 (0.158–1.260)

Multiplicative interaction used the 2×3 LTPA-by-sitting-time product terms. Additive indices compared LTPA <150 vs ≥150 min/week and sitting ≥8 vs <8 h/day; HRs approximate relative risks. The CVD synergy-index denominator was non-positive and therefore not interpretable.

Table S7. CKM-stratum-specific contrast between the least favorable and reference joint categories.

Outcome	CKM stratum	Total n / events	Reference n / events	Target n / events	HR (95% CI)	P	P for heterogeneity
All-cause mortality	Stages 0-1	2161 / 62	455 / 10	379 / 12	1.933 (0.787–4.750)	.151	.958
All-cause mortality	Stage 2	5667 / 341	891 / 38	1270 / 104	1.819 (1.144–2.893)	.011	
All-cause mortality	Stage 3	728 / 267	62 / 17	237 / 107	2.059 (1.070–3.960)	.030	
CVD mortality	Stages 0-1	2161 / 11	455 / 2	379 / 4	2.379 (0.421–13.453)	.327	.402
CVD mortality	Stage 2	5667 / 80	891 / 6	1270 / 31	3.694 (1.236–11.044)	.019	
CVD mortality	Stage 3	728 / 84	62 / 7	237 / 31	1.308 (0.427–4.001)	.638	
Non-CVD mortality	Stages 0-1	2161 / 51	455 / 8	379 / 8	1.770 (0.588–5.327)	.310	.275
Non-CVD mortality	Stage 2	5667 / 261	891 / 32	1270 / 73	1.451 (0.831–2.532)	.190	
Non-CVD mortality	Stage 3	728 / 183	62 / 10	237 / 76	2.862 (1.564–5.236)	<.001	

Target: LTPA <150 min/week with sitting ≥8 h/day; reference: sufficient LTPA with sitting <6 h/day. Stages 0–1 were pooled for sparse cause-specific cells. The survey Cox model was restricted to these two groups, included exposure-by-stratum terms, and adjusted for age, sex, race/ethnicity, education, poverty-income ratio, smoking, current alcohol use, and insurance. Counts are unweighted; heterogeneity P is from the joint design-adjusted Wald test.

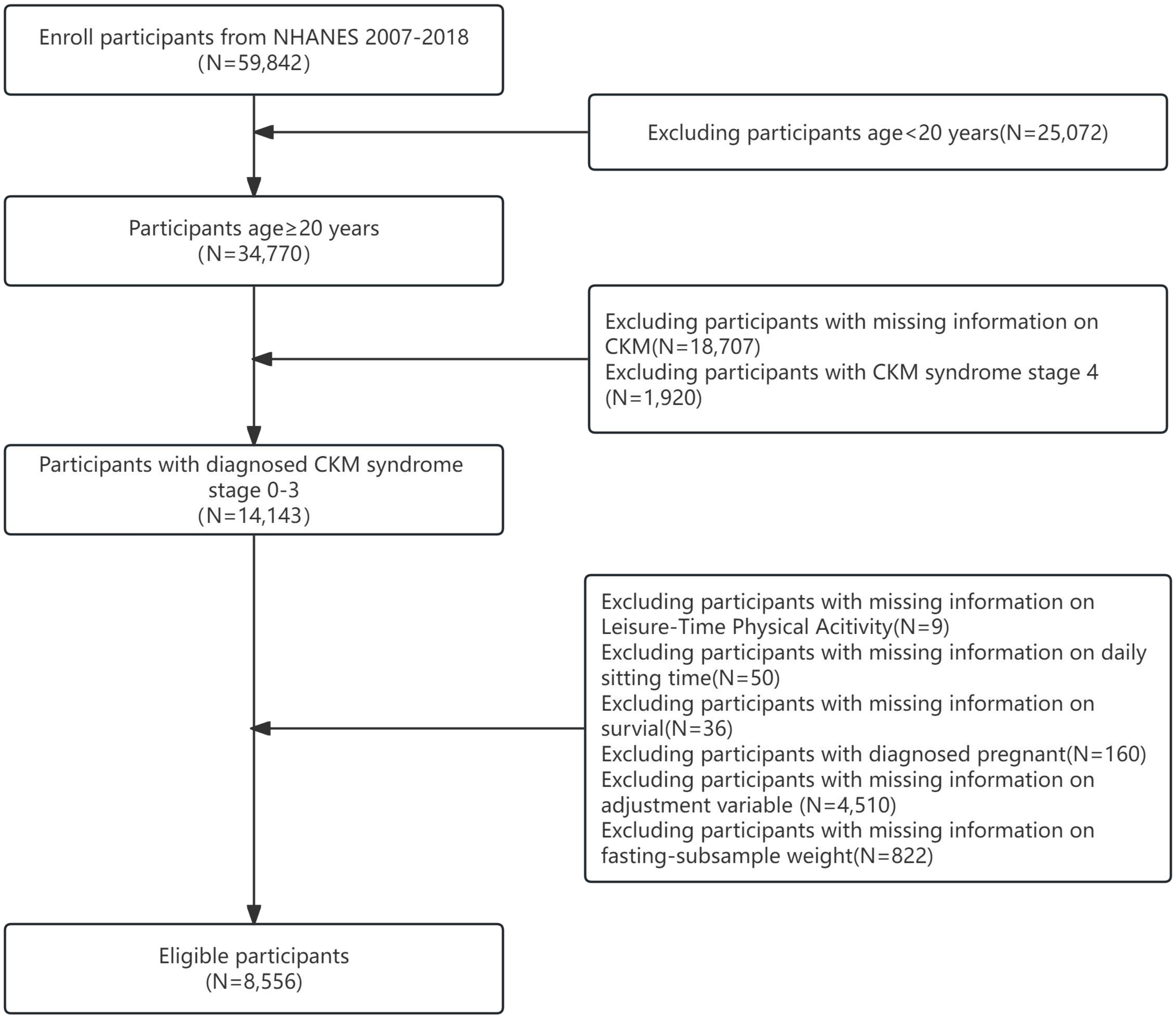

Figure S1. Flow chart for participant selection.

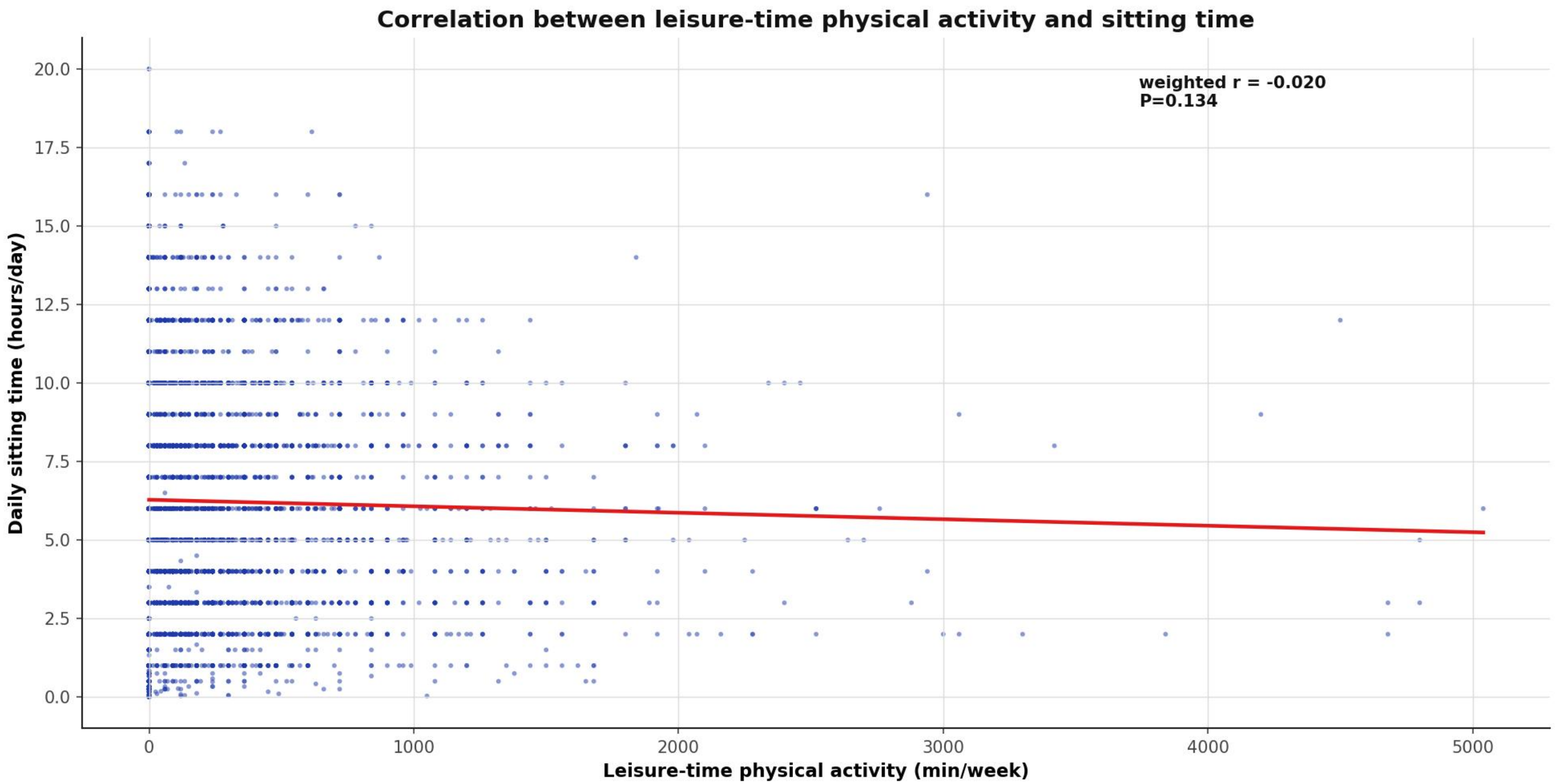

Figure S2. Relation between daily sitting time and leisure-time physical activity in the primary analytic cohort.

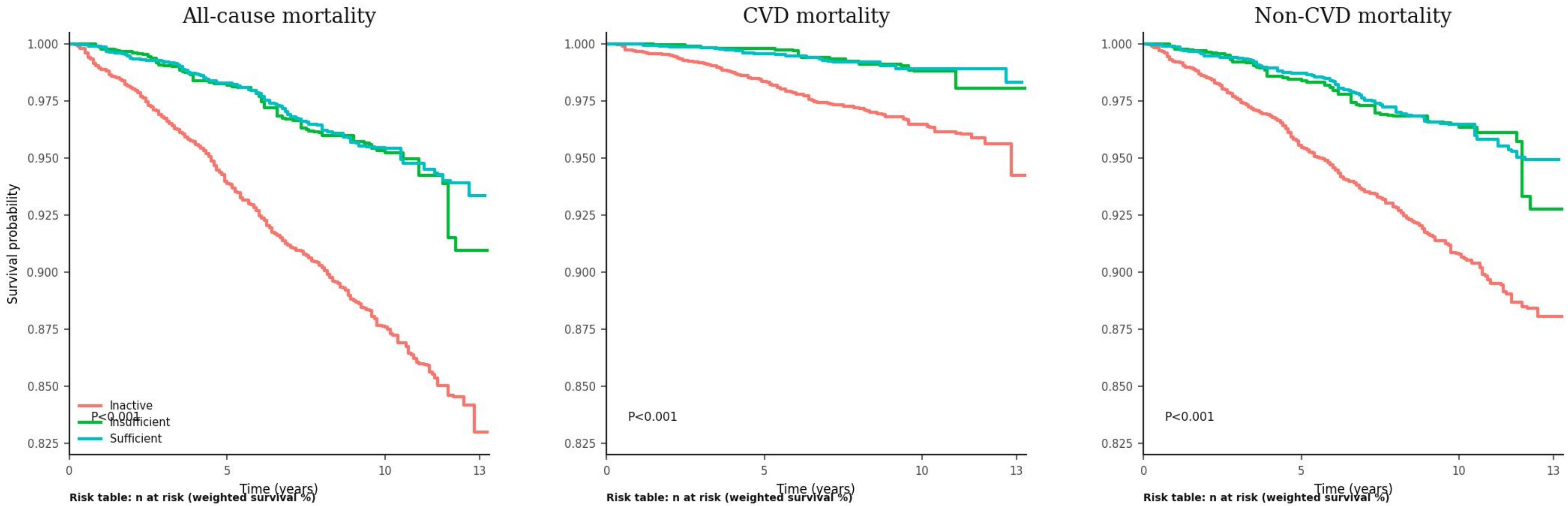

Risk table: n at risk (weighted survival %) — All-cause mortality

	0	5	10	13
Inactive	4536 (100.00)	2906 (93.88)	1094 (87.61)	17 (83.00)
Insufficient	1360 (100.00)	907 (98.21)	322 (95.23)	9 (90.96)
Sufficient	2660 (100.00)	1791 (98.29)	639 (95.42)	14 (93.37)

Risk table: n at risk (weighted survival %) — CVD mortality

	0	5	10	13
Inactive	4536 (100.00)	2906 (98.36)	1094 (96.49)	17 (94.26)
Insufficient	1360 (100.00)	907 (99.80)	322 (98.82)	9 (98.06)
Sufficient	2660 (100.00)	1791 (99.57)	639 (98.92)	14 (98.34)

Risk table: n at risk (weighted survival %) — Non-CVD mortality

	0	5	10	13
Inactive	4536 (100.00)	2906 (95.45)	1094 (90.80)	17 (88.06)
Insufficient	1360 (100.00)	907 (98.40)	322 (96.36)	9 (92.75)
Sufficient	2660 (100.00)	1791 (98.71)	639 (96.47)	14 (94.95)

Figure S3. Survey-weighted Kaplan–Meier curves for all-cause, CVD, and non-CVD mortality, stratified by leisure-time physical activity.

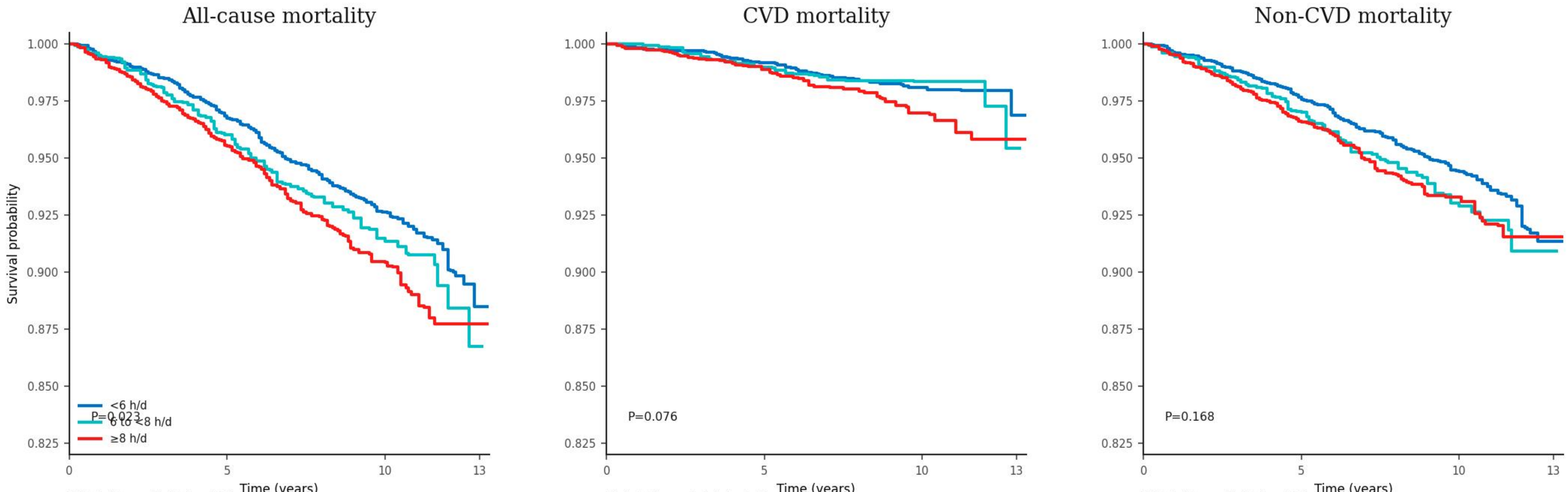

Risk table: n at risk (weighted survival %)

	0	5	10	13
<6 h/d	4510 (100.00)	2947 (96.74)	1239 (92.62)	24 (88.50)
6 to <8 h/d	1300 (100.00)	839 (96.02)	298 (91.37)	4 (86.75)
≥8 h/d	2746 (100.00)	1818 (95.52)	518 (90.44)	12 (87.73)

Risk table: n at risk (weighted survival %)

	0	5	10	13
<6 h/d	4510 (100.00)	2947 (99.17)	1239 (98.09)	24 (96.88)
6 to <8 h/d	1300 (100.00)	839 (98.97)	298 (98.35)	4 (95.43)
≥8 h/d	2746 (100.00)	1818 (98.89)	518 (96.96)	12 (95.83)

Risk table: n at risk (weighted survival %)

	0	5	10	13
<6 h/d	4510 (100.00)	2947 (97.55)	1239 (94.42)	24 (91.35)
6 to <8 h/d	1300 (100.00)	839 (97.01)	298 (92.90)	4 (90.91)
≥8 h/d	2746 (100.00)	1818 (96.59)	518 (93.28)	12 (91.54)

Figure S4. Survey-weighted Kaplan–Meier curves for all-cause, CVD, and non-CVD mortality, stratified by daily sitting time.

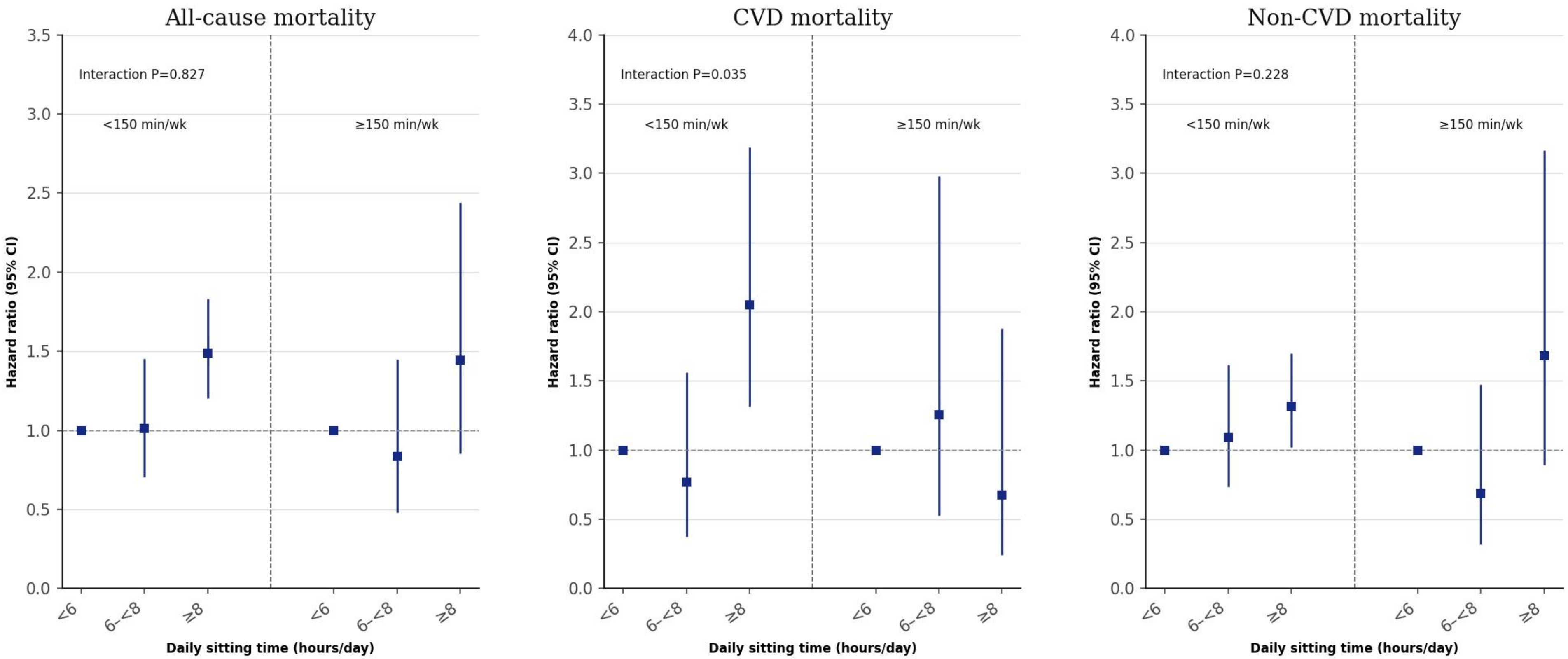

Figure S5. Associations of daily sitting time with mortality among US adults classified as CKM stages 0–3, stratified by leisure-time physical activity.

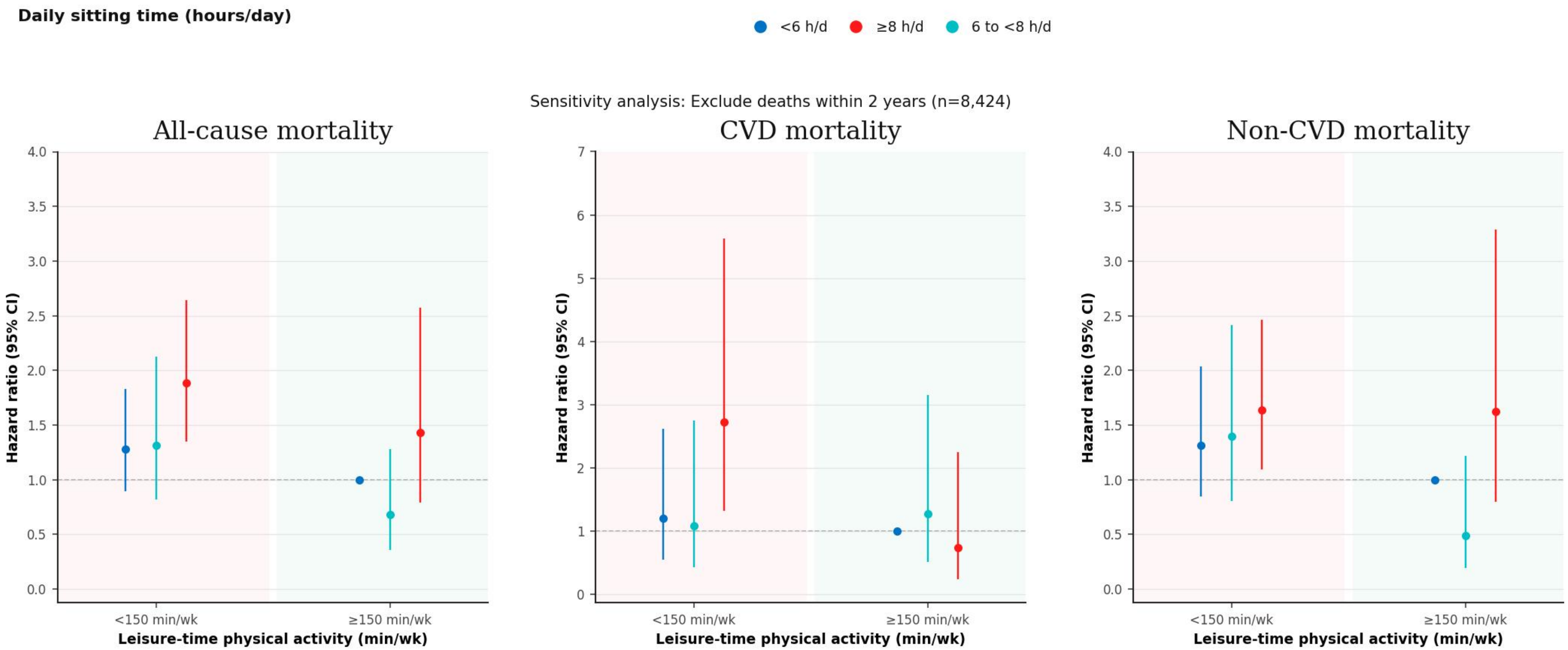

Figure S6. Joint associations of leisure-time physical activity and daily sitting time with mortality after excluding deaths within 2 years.

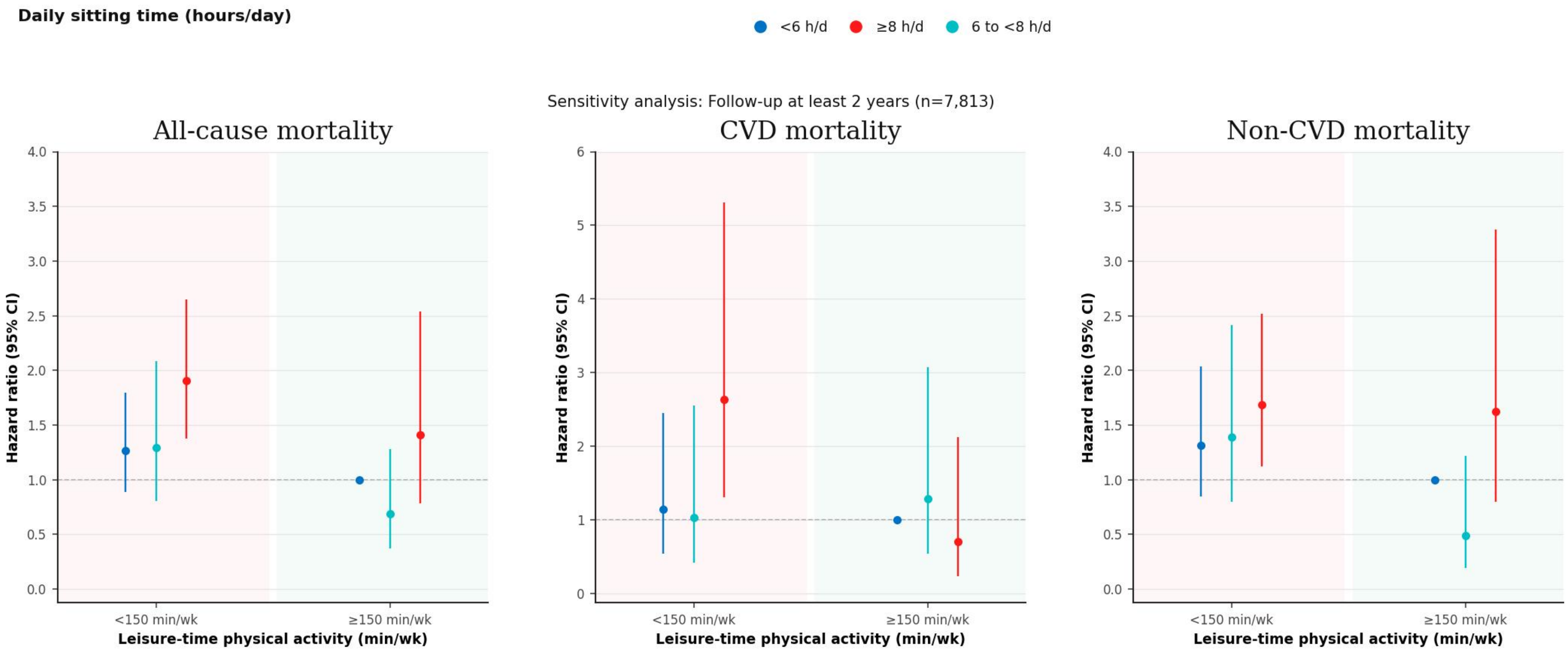

Figure S7. Joint associations of leisure-time physical activity and daily sitting time with mortality among participants followed for at least 2 years.

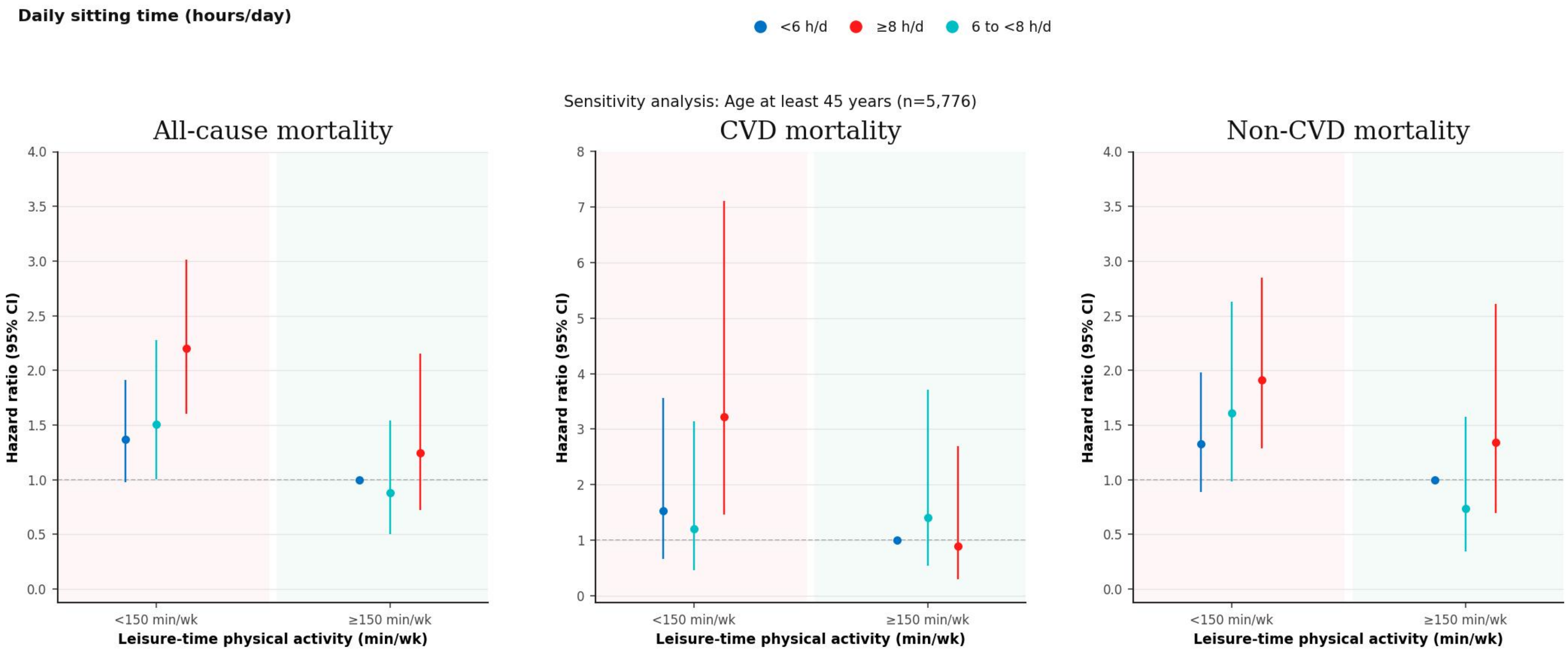

Figure S8. Joint associations of leisure-time physical activity and daily sitting time with mortality among participants aged at least 45 years.

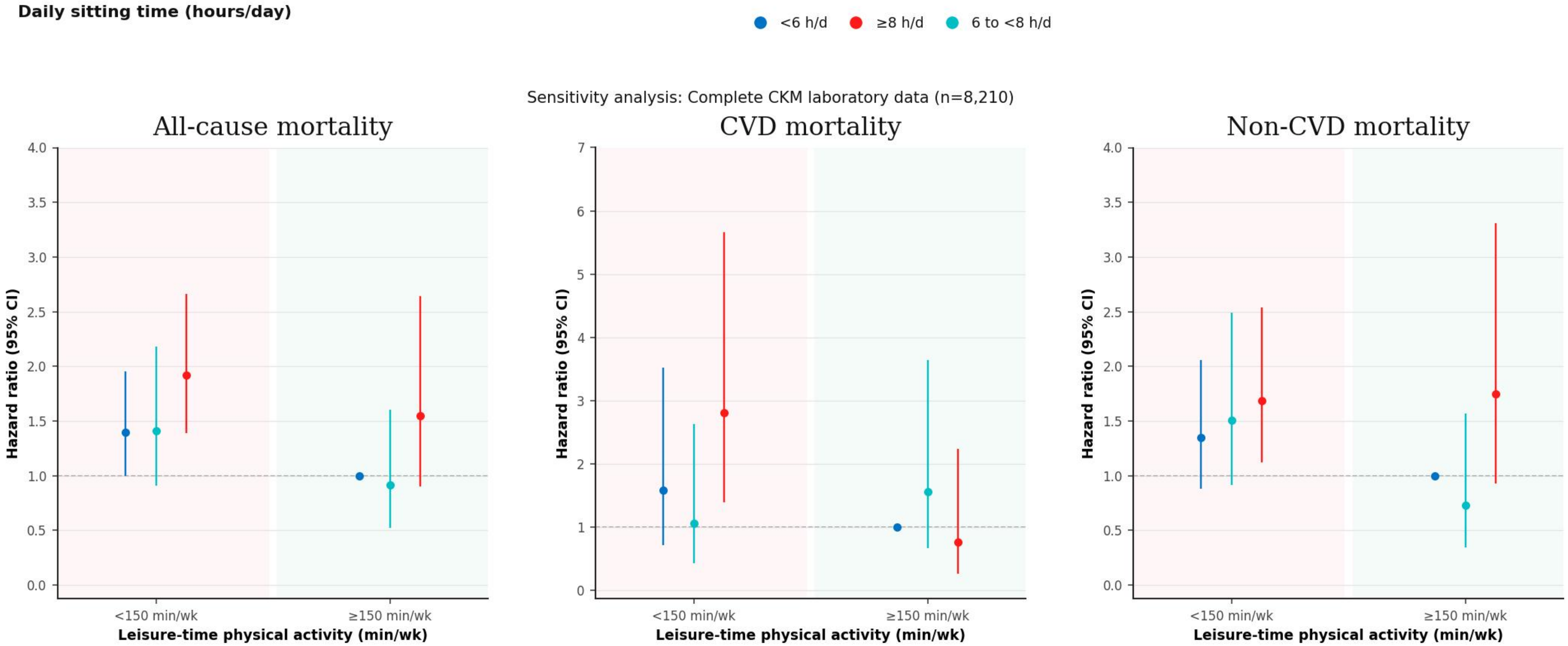

Figure S9. Joint associations of leisure-time physical activity and daily sitting time with mortality among participants with complete CKM laboratory component data.

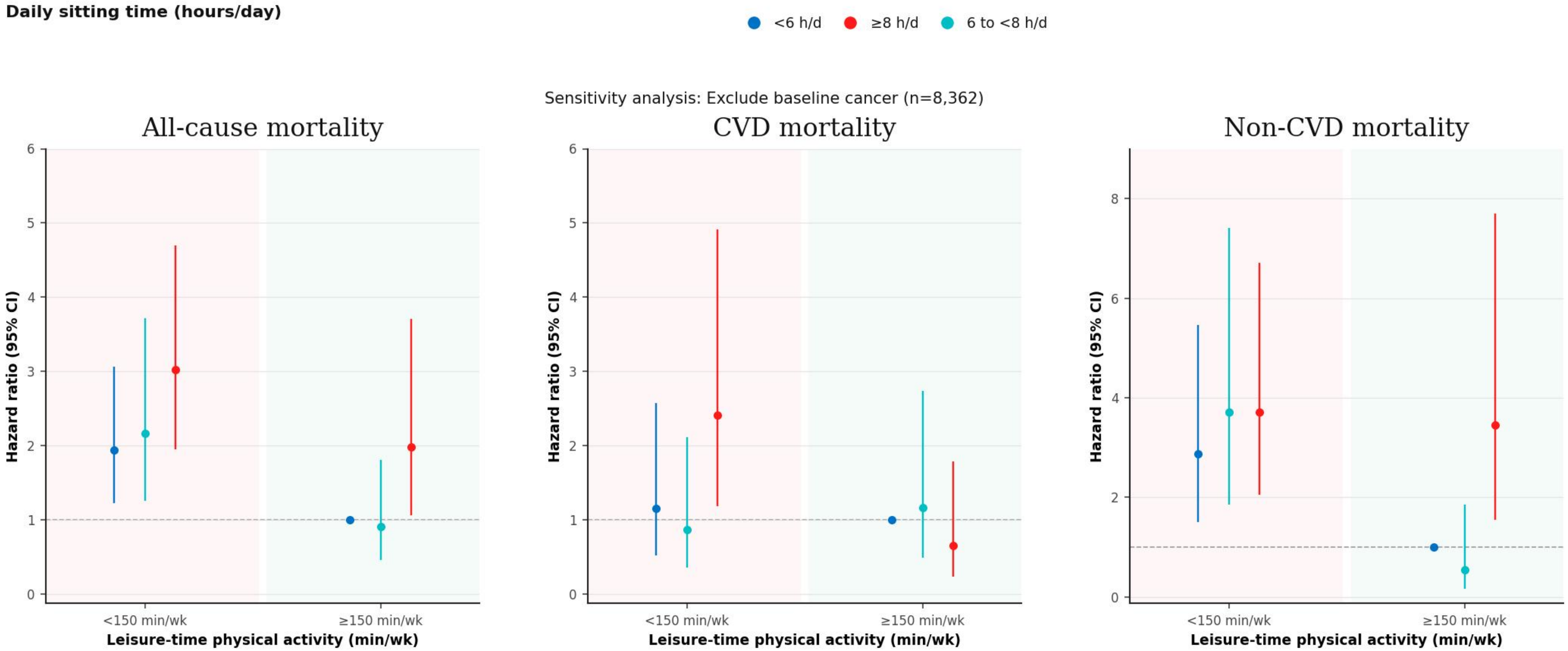

Figure S10. Joint associations of leisure-time physical activity and daily sitting time with mortality after excluding participants with baseline cancer.

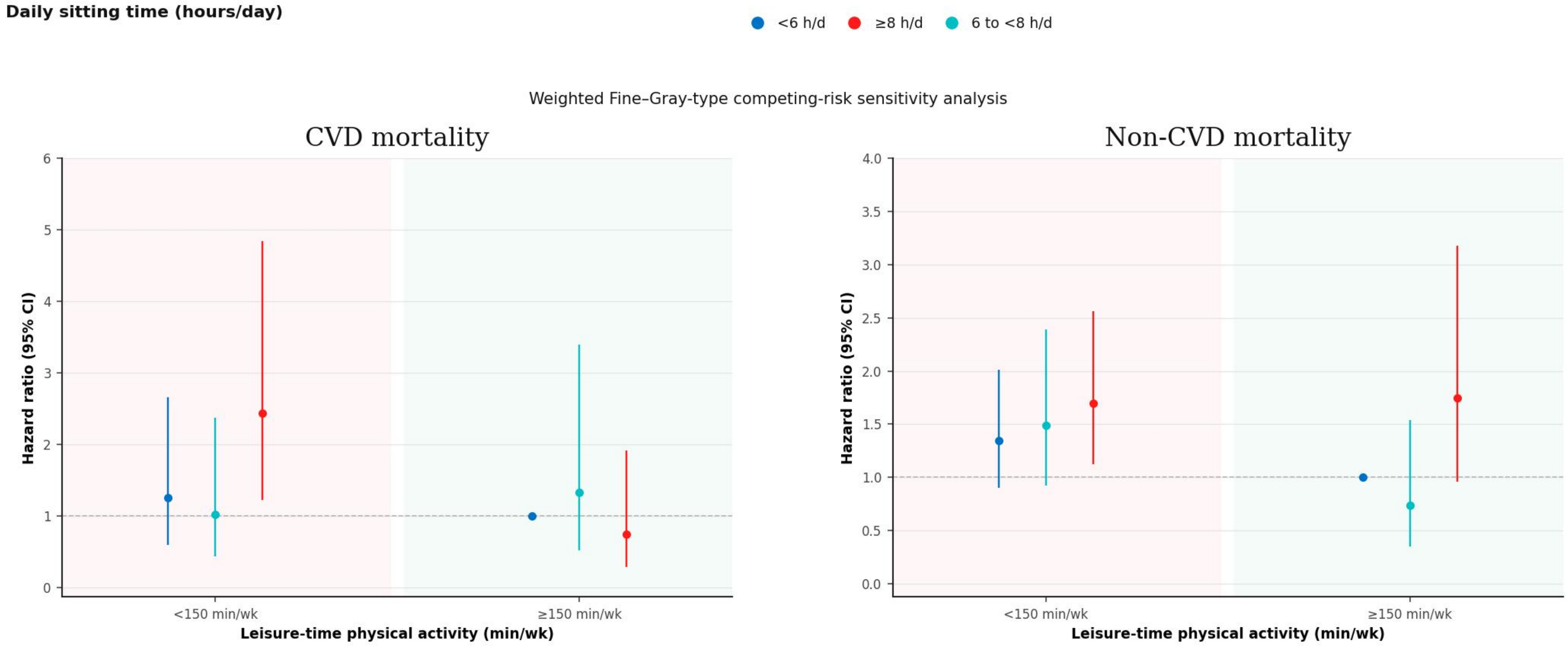

Figure S11. Joint associations of leisure-time physical activity and daily sitting time with CVD and non-CVD mortality in weighted subdistribution hazard models.

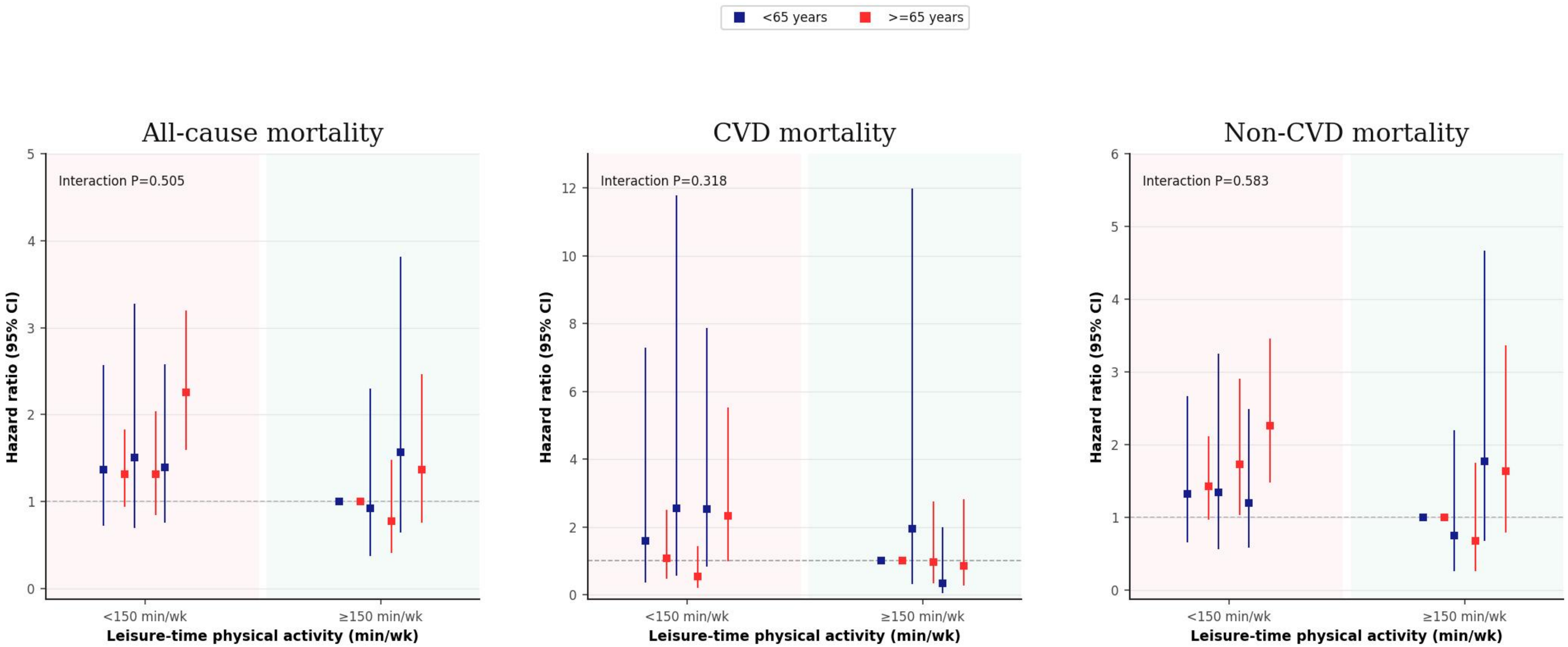

Figure S12. Joint associations of leisure-time physical activity and daily sitting time with mortality, stratified by age.

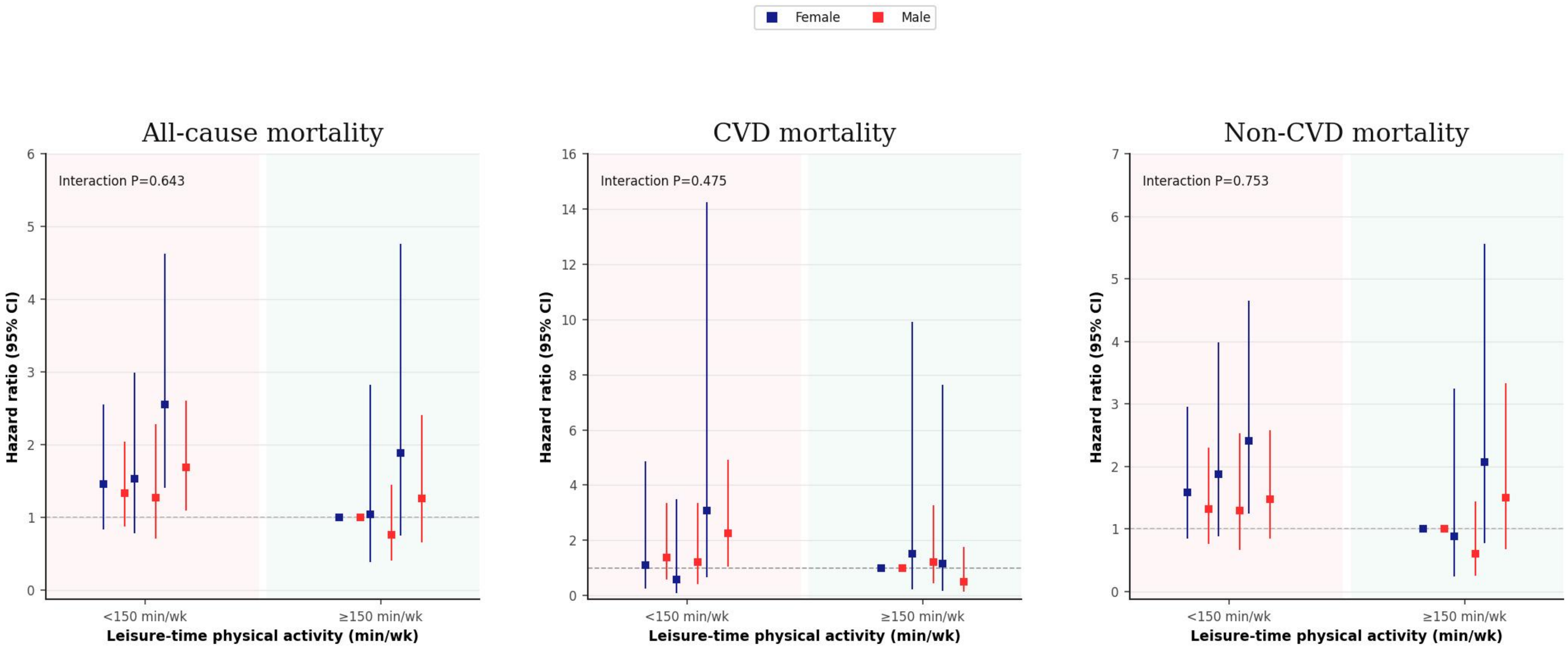

Figure S13. Joint associations of leisure-time physical activity and daily sitting time with mortality, stratified by sex.
